# Data Sharing Reveals Complexity in the Westward Spread of Domestic Animals across Neolithic Turkey

**DOI:** 10.1371/journal.pone.0099845

**Published:** 2014-06-13

**Authors:** Benjamin S. Arbuckle, Sarah Whitcher Kansa, Eric Kansa, David Orton, Canan Çakırlar, Lionel Gourichon, Levent Atici, Alfred Galik, Arkadiusz Marciniak, Jacqui Mulville, Hijlke Buitenhuis, Denise Carruthers, Bea De Cupere, Arzu Demirergi, Sheelagh Frame, Daniel Helmer, Louise Martin, Joris Peters, Nadja Pöllath, Kamilla Pawłowska, Nerissa Russell, Katheryn Twiss, Doris Würtenberger

**Affiliations:** 1 Department of Anthropology, University of North Carolina at Chapel Hill, Chapel Hill, North Carolina, United States of America; 2 Alexandria Archive Institute, Open Context, San Francisco, California, United States of America; 3 D-Lab, University of California, Berkeley, Berkeley, California, United States of America; 4 Institute of Archaeology, University College London, London, United Kingdom; 5 University of Groningen, Institute of Archaeology, Groningen, Netherlands; 6 Cultures et Environnements Préhistoire, Antiquité, Moyen Âge, Université Nice Sophia-Antipolis, Nice, France; 7 Department of Anthropology, University of Nevada Las Vegas, Las Vegas, Nevada, United States of America; 8 Institute for Anatomy, Histology and Embryology, University of Veterinary Medicine, Vienna, Austria; 9 Institute for Prehistory, Adam Mickiewicz University, Poznań, Poland; 10 School of History, Archaeology and Religion, Cardiff University, Cardiff, United Kingdom; 11 Archaeology, Classics and Egyptology, University of Liverpool, Liverpool, United Kingdom; 12 Royal Belgian Institute of Natural Sciences, Bruxelles, Belgium; 13 Department of Anthropology, Stony Brook University, Stony Brook, New York, United States of America; 14 Kingston, Ontario, Canada; 15 Archéorient, Maison de l'Orient et de la Méditerranée, Antenne de Jalès, Berrias-et-Casteljau, France; 16 Department of Veterinary Sciences, Institute of Palaeoanatomy, Domestication and the History of Veterinary Medicine, Ludwig Maximilian University Munich, Munich, Germany; 17 Institute of Geology, Adam Mickiewicz University, Poznań, Poland; 18 Department of Anthropology, Cornell University, Ithaca, New York, United States of America; 19 Institute of Prehistoric and Historical Archaeology, Vienna University, Vienna, Austria; 20 Bavarian State Collection of Anthropology and Palaeoanatomy, Munich, Germany; University College London, United Kingdom

## Abstract

This study presents the results of a major data integration project bringing together primary archaeozoological data for over 200,000 faunal specimens excavated from seventeen sites in Turkey spanning the Epipaleolithic through Chalcolithic periods, c. 18,000-4,000 cal BC, in order to document the initial westward spread of domestic livestock across Neolithic central and western Turkey. From these shared datasets we demonstrate that the westward expansion of Neolithic subsistence technologies combined multiple routes and pulses but did not involve a set ‘package’ comprising all four livestock species including sheep, goat, cattle and pig. Instead, Neolithic animal economies in the study regions are shown to be more diverse than deduced previously using quantitatively more limited datasets. Moreover, during the transition to agro-pastoral economies interactions between domestic stock and local wild fauna continued. Through publication of datasets with Open Context (opencontext.org), this project emphasizes the benefits of data sharing and web-based dissemination of large primary data sets for exploring major questions in archaeology (Alternative Language Abstract S1).

## Introduction

The origins and spread of domesticated animals in the Neolithic of SW Asia represents a watershed moment in human history. Domestic animals had a profound impact on the economic, socio-cultural and biological development of human societies and their arrival in regions outside of their original zone of domestication in the Fertile Crescent region heralded a major turning point in Old World culture histories [1]–[3]. Despite the transformative nature of this process, the initial spread of domestic livestock out of the Fertile Crescent and westward across Turkey is poorly understood [4]–[11]. As a result, the underlying organization of the economies involved in this pioneering stage of early food production remains unclear.

Understanding the spread of Neolithic animal husbandry has been hampered by the slow and incomplete recovery, analysis, publication and dissemination of archaeological datasets. Advances in data dissemination technologies and professional practices can make archaeological evidence both more comprehensive and amenable to more rigorous forms of analysis. As demonstrated by this paper, data sharing and online publication of primary datasets has the potential to dramatically increase the pace of innovation in the discipline by allowing major archaeological questions, such as the origins and spread of domestic animals and husbandry practices [12], to be addressed with larger, more complete datasets than are available through traditional publication practices alone [13].

Recent zooarchaeological work convincingly locates the origins of western Eurasian domesticates including sheep, goats, cattle, and pigs, in the Fertile Crescent region of SE Turkey, northern Syria, northern Iraq and NW Iran during the ninth millennium cal BC [14]–[19]. From this region, archaeological research has traced the expansion of Neolithic economies westward to Cyprus in the ninth millennium cal BC, into the southern Levant and Zagros regions in the eighth millennium, followed by expansions into the Aegean in the early/mid seventh millennium cal BC, and into the Danube basin and along the northern margins of the Mediterranean in the late seventh and sixth millennia cal BC [6], [20]–[24]. Although it is clear that central and western Turkey were major routes for the spread of agricultural technologies into Europe, these regions have historically received less attention than their neighbors [4], [5], and, until recently, faunal evidence associated with the expansion of Neolithic cultures has been limited.

Within central Turkey, early Neolithic communities practicing at least some plant cultivation emerged in the mid to late ninth millennium cal BC [25], [26], more than a millennium after their first appearance in the Upper Euphrates basin, and develop into a distinctive Central Anatolian Neolithic cultural zone [27]. Further west, in the Lakes region of SW Turkey, multiple Neolithic settlements date to the seventh millennium cal BC with the earliest dates suggesting an initial appearance of food producing cultures as early as 7500 cal BC in the Beyşehir region and perhaps as early as 7000 cal BC further west in the vicinity of Lake Burdur [28]–[34]. Although the earliest dates for the Neolithic occupation of the Cilician coast (modern Çukurova) date to the early seventh millennium cal BC, it is likely that the southern coast was already settled by the end of the eighth millennium cal BC [35]. On the western coast, excavations in the Izmir region have uncovered evidence for multiple Neolithic settlements dating to the seventh millennium cal BC [36]–[40], which extend back to an Aceramic phase likely dating to the late eighth millennium cal BC [4], [41]. Finally, in Northwest Turkey the earliest evidence for farming settlements, known as the Fikirtepe culture, dates to the mid to late seventh millennium cal BC [6], [42]–[45]. Thus there is a clear time transgressive pattern of movement of Neolithic settlements from SE to NW Turkey [9], [46].

Although the material culture (especially ceramics) of the early Neolithic settlements in central and western Anatolia has been synthesized to address the complex processes involved in the spread of Neolithic technologies westward across Anatolia [5], [47]–[50] the animal economies of these settlements, especially those in western Turkey, have never been comprehensively assessed within their regional context. In addition, previous attempts at regional syntheses of Neolithic animal economies have focused on published reports rather than shared datasets resulting in major limitations in the quality and quantity of available data [9], [51]. As a result, the economic foundation of the Neolithic expansion into Europe has remained unclear [8].

**Figure 1:**
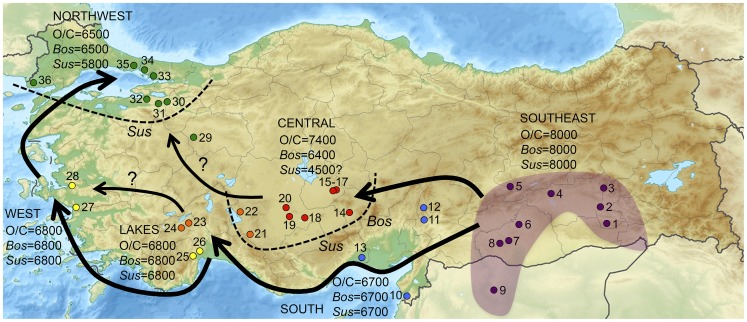
Map of Turkey showing the location of sites mentioned in this analysis. Arrows indicate potential routes for the spread of domestic animals outside of the Fertile Crescent. Dates indicate an approximation of the first appearance of domesticated sheep/goat (O/C), cattle (*Bos*), and pigs (*Sus*) in six regions of Turkey. Dotted lines indicate boundaries where the listed domestic animals were not part of initial Neolithic economies. Southeast Region (purple) = 1. Hasankeyf, 2. Körtik Tepe, 3. Hallan Çemi, 4. Çayönü Tepesi, 5. Cafer Höyük, 6. Nevalı Çori, 7. Göbekli Tepe, 8. Yeni Mahalle, 9. Mureybet; South Region (blue) = 10. Üçağızlı, 11.Domuztepe, 12.Direkli Cave, 13.Yumuktepe; Central Region (red) = 14. Köşk Höyük, 15. Aşıklı Höyük, 16. Musular, 17. Güvercinkayası, 18. Pınarbaşı, 19. Çatalhöyük, 20. Boncuklu; Lakes Region (orange) =  21. Suberde, 22. Erbaba, 23. Höyücek, 24. Bademağacı; West/Coast Region (yellow) =  25. Karain B, 26. Öküzini, 27. Çukuriçi, 28. Ulucak; Northwest Region (green) =  29. Orman Fidanlığı, 30. Barcın, 31. Menteşe, 32. Ilıpınar, 33. Pendik, 34. Fikirtepe, 35. Yenikapı, 36. Hoca Çesme.

To address this problem, and taking advantage of newly available data, this project merges together primary datasets from eighteen researchers into a single database representing seventeen sites (Fig. 1; Table S1), 42 chronological phases, and more than 200,000 faunal specimens in order to provide a comprehensive synthesis of evidence for the tempo and character of the spread of domestic animals westward outside of the Fertile Crescent region of SW Asia. We examine evidence for animal exploitation in Turkey across six phytogeographic zones [52], including the Southeast, Central, Lakes (SW), South, West, and Northwest regions (see Fig. 1; Table S1).

## Materials and Methods

### Data sharing and publication

One of the key scientific contributions of this study centers on the online publication of well documented, standards-aligned, and “cleaned” (edited) datasets which can be used to facilitate replication of the analytical claims made in this paper and provide a foundation for future research further refining our understanding of the spread of Neolithic technologies. The eighteen primary participants in the project agreed to publish their full faunal databases online in the open access, peer reviewed data publishing system Open Context (http://opencontext.org). In keeping with current norms in scientific data curation practices, Open Context archives datasets with the California Digital Library, a university digital repository. All datasets received DOIs (persistent identifiers) and specific metadata documentation to help make them citable resources [53]–[67] (for links to data see Table S1; File S1). These edited datasets can be used to replicate analytical claims made in this paper and in future studies [68]. Details of the approaches used in data cleaning, documentation, semantic annotation and collaboration with data contributors are described elsewhere [69] (see Text S1).

### Zooarchaeological Methods

From these shared databases we report on analyses of three combined datasets including 1) species frequencies, 2) biometrics, and 3) survivorship or age at death data for *Ovis aries/orientalis*, *Capra hircus/aegagrus*, *Bos taurus/primigenius* and *Sus scrofa*. The relative abundance of these taxa was calculated based on NISP (number of identified specimens) identified to the genus level. Because the assemblages used in the study differ in size, were recovered using different methods and were analyzed by researchers utilizing different practices regarding the level of taxonomic identification, we focused on the frequencies of the most abundant large mammal taxa.

Biometric data were recorded following standard protocols [70]. Given the small size of some assemblages, in order to compare breadth and depth measurements from multiple skeletal elements we use the Logarithmic Size Index (LSI) as a generalized measure of body size [71]. The LSI is calculated by transforming measurements from archaeological specimens into their common logarithms and then taking the difference between the resulting value and the logarithm of the same measurement from a standard animal. For our standard animals we utilize four published and widely used individuals including 1) a recent wild female mouflon (*Ovis orientalis*) from Iran [72]; 2) average measurements of a recent male and female bezoar goat (*Capra aegagrus*) from the Taurus mountains [72]; 3) the Ullerslev cow aurochs (*Bos primigenius*) from Mesolithic Denmark [73]; and a recent female wild boar (*Sus scrofa*) from eastern Turkey [74] (for LSI data see File S2).

Mean values and standard deviations were calculated for each taxon (*Ovis, Capra, Bos, Sus*) from each phased assemblage based on the LSI and were statistically compared using an ANOVA and Tukey Honestly Significant Difference tests (see Tables S3–S10). Although differences in the allometry of ancient populations and the standard animal are a source of variation using this method, LSI is a widely used and effective way to identify broad changes in body size at the population level [14], [20], [71], [75]–[77].

Survivorship data were calculated based on the state of fusion of the epiphyses of long bones. Incorporating age data based on tooth eruption and wear proved difficult as a result of multiple and idiosyncratic recording methods used by various database authors and were therefore not incorporated into this analysis. In particular, we focus on the statistic of *% juvenile* which represents the frequency of specimens with unfused long bone epiphyses out of the total sample of fused and unfused specimens. Although long bone epiphyses fuse at a range of ages in bovids and suids (from c. 0–3 months to 48+ months [78]–[80]), the majority of specimens represent skeletal parts that fuse between 12–24 months. The statistic % juvenile is, therefore, a useful general proxy for monitoring changes in the frequencies of skeletally mature versus immature individuals in an assemblage and therefore age of slaughter. Differences in the % juvenile among assemblages representing wild and domestic populations (as evidenced by biometric data and the work of previous authors; see Fig. S1) were statistically compared using Yates Chi Square tests corrected for continuity for each taxon. In addition, Spearman rank order correlation coefficients (r_s_) were calculated in order to address the relationships between % juvenile and %NISP, % juvenile and mean LSI, and between % juvenile and time (years cal BC) for each taxon (Table S11).

### Identifying domesticated animals

The domestication of livestock in Neolithic SW Asia took place over several millennia and involved a combination of cultural and biological processes including the application of intentional management strategies (e.g., control over movement, foddering, young male culling) and the reproductive isolation of managed herds from their wild ancestors [81]–[83]. These processes, which likely began in the tenth millennium cal BC, resulted in the appearance of ‘domesticated’, phenotypically distinctive, husbanded animal populations by the mid to late ninth millennium cal BC in the Fertile Crescent region [14], [17]. In central and western Turkey, where domesticated animals first appear in the eighth and seventh millennia cal BC, respectively [19], [76], [84], three lines of evidence are widely employed to distinguish the management of domestic livestock populations from the hunting of wild populations including 1) changes in body size; 2) demographic evidence for intentional herd management; and 3) appearance of taxa outside of their natural range. Although identifying the nature of the earliest Neolithic animal management strategies in central Turkey still poses considerable challenges [19], these lines of evidence provide a clear picture of the westward spread of domestic animals in the later Neolithic (i.e., seventh millennium cal BC).

**Figure 3:**
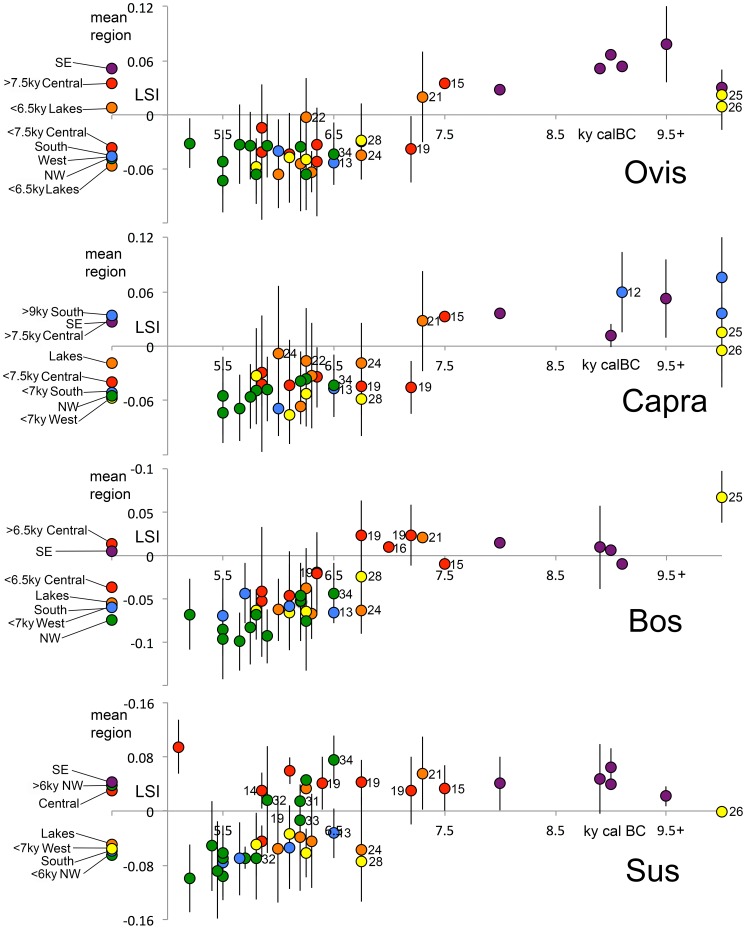
Changes in mean size through time for *Ovis*, *Capra*, *Bos*, and *Sus* (based on LSI of breadth and depth measurements)(for data see Tables S3–6). Vertical lines represent standard deviations. Colors reflect geographic location of site (after Fig. 1). Values to the left of the vertical axis represent means for each region. Key sites are labeled (after Fig. 1).

Measurements of the breadth and depth of long bone epiphyses and some podial bones (e.g., talus, calcaneus) can be used to identify the decrease in body size and reduction in sexual dimorphism commonly associated with the later stages of the domestication process in sheep, goat, cattle and pig [14], [77], [85]–[87]. Although the relationship between body size, animal status (wild versus domestic), and human management is complex, especially in the early stages of the domestication process [88]–[90], change in body size is a useful proxy for identifying the spread of domesticated animal populations especially in the later Neolithic when they exhibit significant phenotypic changes compared to their wild ancestors (Fig. 3) [91]. In this study the term ‘domesticated’ is used to refer to animals exhibiting phenotypic changes associated with long-term, intensive human management. It is therefore distinguished from the long-term ‘domestication process’, during the early stages of which phenotypically wild animals came under human management. Biometric data from earlier Neolithic sites in SE Turkey representing morphologically wild populations of sheep, goat, aurochs and boar were used to provide a baseline for identifying changes in body size in later central and western Anatolian Neolithic populations. Although it is acknowledged that it would be preferential to use local wild populations in each subregion of Anatolia as a benchmark for defining size change [19], this is not possible with currently available datasets.

Animal husbandry regimes frequently involve the systematic culling of specific demographic groups within a herd [87]. These practices, which often target young males not needed for herd reproduction, result in higher frequencies of juveniles in archaeofaunal assemblages compared to most hunting practices and can therefore be used to identify systems of animal husbandry [92]. This is particularly true for sheep and goats where it has been used to identify early husbandry strategies [15], [88] but is also relevant for cattle and pigs—although swine management strategies exhibit more variability due to the multiparous nature of this species [93].

Finally, the appearance of taxa outside of their natural habitat may suggest that they were transported under conditions of human management [87], [94]. The natural range of wild sheep and goats includes the mountains and plains of eastern, south-central, and southwestern Turkey [95]. Western and NW Turkey are outside of this range and the appearance of these taxa in these regions indicates that they were introduced as domesticates. This line of evidence is less useful for *Sus* and *Bos*, which have less restricted early Holocene ranges. Wild boar is currently found in every province of Turkey and aurochs are expected to have had an equally wide distribution in the early Holocene [51], [76]. In combination, biometric evidence for size change, increased juvenile culling, and the appearance of taxa outside of their natural habitat provide a reliable means to identify domestic animals and animal husbandry in Neolithic archaeofaunal assemblages in central and western Anatolia dating from the eighth to the sixth millennia cal BC.

## Results

### Regionalization and species preferences

**Figure 2:**
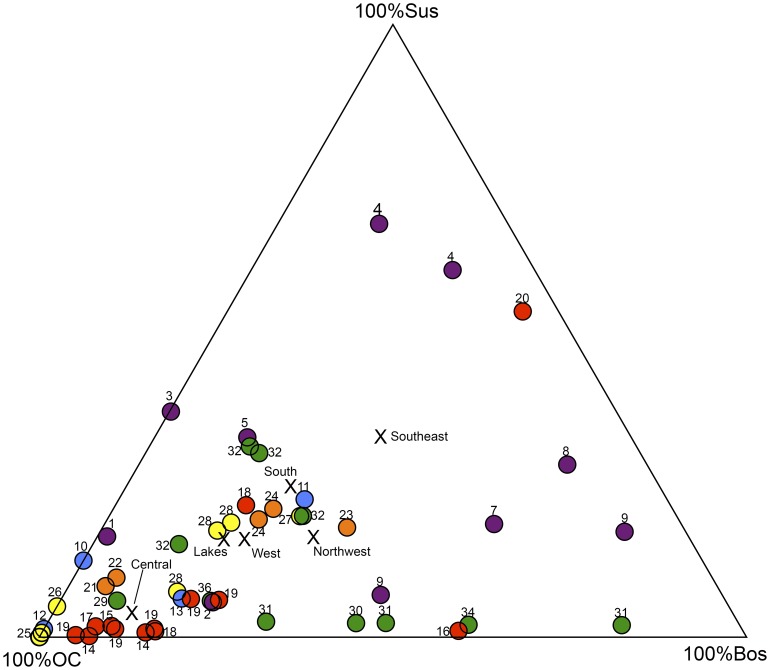
Relative frequencies of OC (*Ovis aries/orientalis*+*Capra hircus/aegagrus*), *Bos taurus/primigenius*, and *Sus scrofa* in Neolithic assemblages (see Table S2 for data). Sites labeled and colored after Fig. 1. X's represent mean values for each region.

As animal economies based on the management of domestic livestock moved outside of the Euphrates and Tigris basins where they were characterized by highly variable combinations of caprines (i.e., sheep [*Ovis aries*] and goats [*Capra hircus*]), cattle (*Bos taurus*), and pigs (*Sus scrofa*) (Fig. 2; Table S2), they took on regional characteristics in response to local of ecogeographic conditions and cultural preferences [8], [9], [19], [76], [96]. The earliest assemblages examined in this study indicate that wild sheep and goats were intensively targeted by Epipaleolithic hunters in the Taurus mountains at Karain B, Öküzini and Direkli Caves. In central Turkey the earliest Neolithic economies include large numbers of wild boar, as seen at Pınarbaşı A and Boncuklu [25]. However, with the exception of the site Musular [97], where aurochs were heavily exploited [75], all later Neolithic animal economies in this region focused intensively on caprines (central Turkey mean_caprineNISP_ = 87%) with modest amounts of *Bos* and very little *Sus* (Fig. 2; Table S2). In the Lakes region and western Turkey, although caprines are abundant, pigs and cattle represent larger portions of the animal economies (Lakes region mean_Bos+SusNISP_ = 32%; western Turkey mean_Bos+SusNISP_ 34%)(Fig. 2;Table S2). This is also the case in southern Turkey where cattle and pigs together nearly match caprines in abundance (South Turkey mean_Bos+SusNISP_ = 48%) (Fig. 2;Table S2). Finally, in the NW region cattle increase to their highest levels (NW Turkey mean_BosNISP_ = 29%)—a trend that has been linked with an increase in the consumption of dairy products [51], [98].

### Biometric evidence for domesticates

Biometric data has long been used to identify changes in phenotype associated with the process of animal domestication [85], [87], [93]. In Neolithic Turkey, biometric data for sheep and goats each show a sharp decrease in body size (compared to late Pleistocene/early Holocene wild caprines), which is first evident in the second half of the eighth millennium cal BC at the site of Çatalhöyük, central Turkey (Fig. 3; Tables S3–S4; for statistical comparison see Tables S7–S8). This represents the first appearance of domesticated (i.e., exhibiting a domestic phenotype) caprines in Turkey outside of the Fertile Crescent region. Although not evident at the early Neolithic site of Suberde, small-sized, domesticated sheep and goats appear in the Lakes region and in western and southern Turkey in the early seventh millennium cal BC and in NW Turkey in the mid to late seventh millennium cal BC (Fig. 3; Tables S3–S4).

In the Lakes region the mean size of goats at Erbaba, Bademağacı, and Höyücek is notably larger than that for contemporary sites in central, western and NW Turkey, while for sheep the Erbaba and Çatalhöyük West assemblages are significantly larger than all other later Neolithic assemblages and even overlap with the wild caprines from late Pleistocene Karain B and Öküzini caves (Fig. 3; Table S3–S4, Tables A–E in File S1). All of these sites are located within the natural habitats of wild sheep and/or goats, and it is possible that this pattern reflects hunting and/or interbreeding with local wild caprine populations by Neolithic agro-pastoralists in these regions.

For *Bos*, a dramatic decrease in body size compared to Anatolian aurochs is evident in the early seventh millennium cal BC in the Lakes region at Bademağacı ENI and in western Anatolia at Ulucak VI (Fig. 3; Tables S5, S9). Small sized, domestic cattle appear later in central Turkey where they are only evident in the later levels (South P-T and TP Area) at Çatalhöyük dating to the mid to late seventh millennium cal BC. At the same time, domestic cattle also arrive with the earliest Neolithic settlements in NW Turkey where they exhibit the smallest mean size for any region [76], [91], [99].

Patterns of size change for suids differ from those observed in other taxa. Surprisingly, the earliest small-sized domestic pigs appear in western Turkey at Ulucak VI in the early seventh millennium cal BC and they are evident slightly later in the Lakes region at Bademağacı and at Yumuktepe in southern Turkey (Fig. 3; Tables S6, S10). Our data indicate that domestic pigs were never incorporated into Neolithic economies in central Turkey, where *Sus* remains are rare and comparable in size to early Holocene wild boar into the fifth millennium cal BC. The only exception to this comes from two specimens from the latest deposits (West Mound) at Çatalhöyük that are within the size range of Neolithic Anatolian domestic pigs (Fig. 3; Table S6). Given the extremely low frequency of *Sus* in these levels (<1% total NISP) and the lack of juveniles, these specimens may represent gifts or imported oddities rather than evidence for onsite pig husbandry. Alternately, it is also possible that they represent particularly small wild boar or intrusive specimens representing domesticates from later periods. Domestic pigs are also notably absent in the earliest Neolithic settlements in NW Turkey including Ilıpınar X, Barcın VI, Fikirtepe, Menteşe ancien, and Hoca Çesme. However, they appear suddenly in the NW after 6000 cal BC as seen at Ilıpınar IX, Menteşe récent, and Yenikapı.

### Age at death

**Figure 4:**
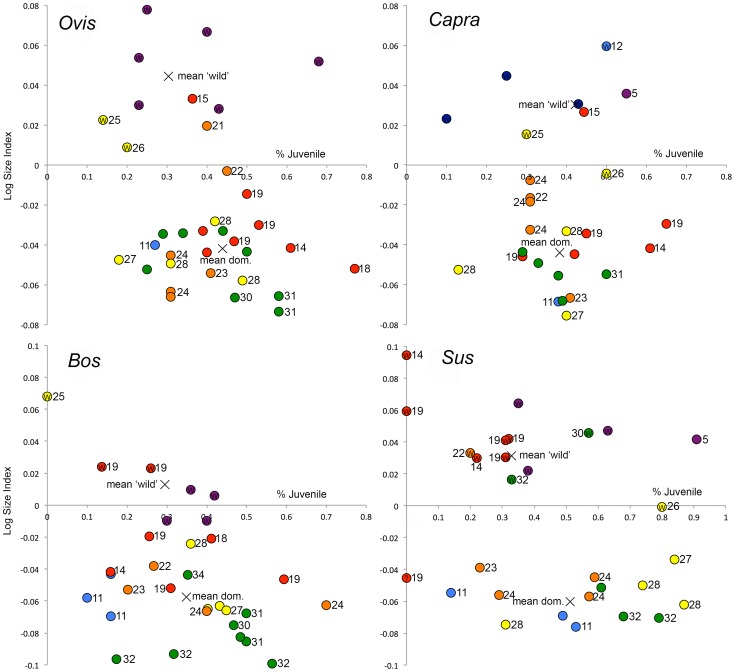
Plots showing relationship between body size (mean LSI) and %juvenile (based on long bone fusion) for *Ovis*, *Capra*, *Bos*, and *Sus* (for data see Tables S3–6). Colors reflect geographic location of site (after Fig. 1). “W” indicates assemblages representing wild populations. Key sites are labeled (after Fig. 1). For *Capra*, dark blue marks represent Zagros sites Asiab, ZC Shanidar, and Shanidar Mousterian.

Age at death data combined with biometrics (Fig. 4) provide strong supporting evidence that Neolithic bovids and suids exhibiting a domestic phenotype were under intensive human management. Neolithic assemblages in which small sized domesticates are present exhibit increased juvenile culling compared to those dominated by phenotypically wild bovids and suids—a pattern that fits the expectations of models for early herd management [14], [87], [100], [101] (Table S11). For *Ovis*, *Bos* and *Sus* the frequency of juveniles is significantly higher in assemblages with small-sized domesticates compared to those representing phenotypically wild, hunted populations, clearly reflecting the effects of intentional human management strategies (Chi-square test: *Ovis*, X^2^ = 21.64, df = 1, p<0.0001; *Bos*, X^2^ = 6.38, df = 1, p = 0.0115; *Sus*, X^2^ = 25.06, df = 1, p<0.001). For example, in central Turkey the first appearance of domestic cattle in the mid seventh millennium cal BC corresponds with an increase in the frequency of juvenile individuals from 13% to 35%. As a result of the intensive culling of juvenile goats in some Epipaleolithic and early Neolithic (PPNA, EPPNB) assemblages, *Capra* is the exception to this pattern showing no change in the frequency of juveniles among assemblages representing domestic versus wild animals (X^2^ = 0.3, df = 1, p = 0.5839).

More generally, *Ovis* and, to a lesser extent, *Bos* both exhibit steady increases in the frequencies of juveniles over time across all regions (Fig. S1). Assemblages with the highest frequencies of domestic sheep (those in central Turkey) and domestic cattle (those in NW and western regions) also exhibit the most intensive juvenile culling (Tables S2, S3, S5, S11). For *Capra* and *Sus*, which show more demographic variability in assemblages produced by hunting, there are no overall changes in the representation of juveniles over time, although assemblages with domestic pigs exhibit more intensive juvenile culling (mean_juv_ = 51%) than those representing wild boar (mean_juv_ = 32%) (Fig. 4; Fig. S1; Tables S2, S4, S6, S11).

## Discussion

### Chronological trajectories for the spread of domestic animals

The patterns emerging from the shared datasets used in this project suggest that the movement of domesticated animals and husbandry techniques westward across Neolithic Turkey followed several different trajectories, clearly indicating that livestock did not move as part of a standard agro-pastoral ‘package’ [50]. Although domestic sheep and goats appear first in central Turkey by the mid eighth millennium cal BC, domestic cattle appear a millennium later and domestic pigs are never incorporated into Neolithic economies in this region [76]. The combination of domestic caprines, cattle and pigs occurs for the first time in Turkey, outside of the SE, in the early seventh millennium cal BC, where all four livestock species are evident in the Izmir region at Ulucak VI and then slightly later in SW Turkey at Bademağacı ENI, and in southern Turkey at Yumuktepe [102] (Fig. 1). Since a completely autochthonous domestication event in western Turkey is unlikely, this pattern suggests a rapid westward movement of domestic animals across southern Turkey [4]. The geography and timing of this early expansion suggest it may have followed a coastal route, either by land or by sea [20], although the initial phases of this process in southern Turkey are currently poorly documented [40], [103]. However, the absence of domestic cattle and pigs in central Turkey at this time suggests that this region was not directly involved in the earliest spread of domestic livestock either to the Lakes region or western Turkey and reaffirms the distinctive nature of the Central Anatolian Neolithic tradition [104]–[106].

Finally, the earliest Neolithic communities in NW Turkey utilized domestic caprines and cattle—with a marked increase in the latter including a new emphasis on culling juveniles—but did not keep domestic pigs [8], [76]. This suggests that the origin of the Neolithic in the NW was not a simple extension of the same colonization processes that brought farming communities to SW and western Turkey but instead represents a distinctive process, perhaps involving different populations of farmers and/or interaction with local foragers [47], [49], [107]. The initial absence of pig husbandry at sites pertaining to the archaic Fikirtepe tradition in NW Turkey may reflect central Anatolian influences, which have been noted in the early Neolithic material culture in this region, although they may also reflect responses to local environmental and economic conditions [6]. In either case, the adoption of pig husbandry in the early sixth millennium cal BC in the NW suggests increasing interaction with neighboring groups along the Aegean coast where pig husbandry had been practiced for almost a millennium [4]–[6], [8], [76], [84].

### The spread of domestic animals and Neolithic traditions

The broad patterns evident in the faunal data can be linked with recent interpretations of material culture evidence for the spread of Neolithic technologies in western and NW Turkey. It has been argued that the westward spread of farming across Turkey was characterized by multiple distinctive impulses [4]–[6], [42]. The first of these relates to the westward movement of an Aceramic tradition (contemporary with the Levantine Late PPNB) with Near Eastern features including red plaster floors, known primarily from Ulucak VI, Bademağacı and Aceramic Hacılar and dating to the late eighth and early seventh millennia cal BC [4], [6], [34], [41]. Subsequent impulses involve the movement of two distinctive ceramic traditions including first, the overland expansion of the so-called Dark Faced Burnished Ware (DFBW) tradition westward and into the Marmara region in the mid seventh millennium, followed by the slightly later movement of the Red Slipped Burnished Ware (RSBW) horizon along the coast and into interior SW Turkey [5], [6], [42], [44], [47], [107], [108].

The faunal patterns identified in this project fit broadly within these distinctive Neolithic material culture traditions. The movement of domestic caprines, cattle and pigs along the southern and western regions of Turkey evident at Ulucak VI, and slightly later at Bademağacı ENI and Yumuktepe corresponds with the initial Aceramic expansion of Neolithic lifeways and with the later RSBW horizon. The presence of convincing material culture parallels between the early settlements in these regions as well as similarities in their animal economies supports the presence of a distinctive late eighth-seventh millennium cal BC coastal, and perhaps inland, social network linking southern, SW and western Turkey [4], [5], [11], [37], [48], [107], [109].

In contrast, the movement into NW Turkey of a distinctive animal economy characterized by domestic caprines and cattle but the absence of domestic pigs may be associated with the spread of the distinctive DFBW tradition from the interior Anatolian plateau. Here similarities between the Dark Burnished wares from central Turkey and the archaic Fikirtepe tradition are supported by a shared absence of domestic pigs, a distinctive feature of both regions, as well as the shared exploitation of dairy products and other well documented material culture correlates [5], [6], [11], [76], [98], [110], [111]. In addition, domestic cattle first appear in central Turkey in the mid seventh millennium cal BC, approximately contemporaneous with their introduction into the Marmara region where they become the focus of the animal economy.

### Incorporation of local wild fauna into domestic herds

Our data also shed light on processes that took place during the western spread of Neolithic domestic animals. In the Lakes region of SW Turkey, goats exhibit significantly larger mean sizes compared to Neolithic goats from other regions; the same pattern is evident for the sheep from Erbaba. Moreover, in central Anatolia, following the appearance of domesticates in the mid seventh millennium cal BC, cattle exhibit mean sizes larger than those for any other region of Anatolia examined in this study (including SW, West, South, NW). Neolithic settlements in central and SW Turkey are located within the natural habitat of wild sheep, goats, and aurochs, and it is suggested that these patterns in the biometric data reflect intensive interaction between Neolithic human populations and local wild fauna [32]. Although it is possible that the biometric data simply reflect the opportunistic hunting of locally available bezoar, mouflon, and aurochs, it is also possible that these patterns reflect the intentional (or accidental) incorporation of local wild animals into domestic livestock populations.

For cattle, it has been suggested that intentional breeding of domesticate cows with bull aurochs may have been a regular part of a ritual-laden management strategy at Çatalhöyük [112]. Moreover, recent paleogenetic work on suids in Turkey has shown that the distinctive maternal lineage of domestic pigs involved in the initial Neolithic expansion into Europe, likely derives from western/SW Anatolian boar populations [103]. This suggests that Neolithic communities in western Turkey incorporated local wild sows into their domestic herds which were then introduced into Neolithic Europe in the late seventh millennium cal BC [103], [113]. In light of this genetic evidence for the incorporation of local boar into domestic herds, combined with biometric evidence for interaction with wild sheep, goats, and aurochs, it can be hypothesized that SW (and perhaps also central) Turkey represents a particularly important area of interaction between humans, domestic animals and wild populations where the genotype and phenotype of Neolithic livestock populations were altered prior to expansions westward into Europe. Paleogenetic studies of caprines and also cattle from sites in this region dating to the eighth and seventh millennia cal BC are necessary to explore this issue further.

## Conclusion

Using shared primary faunal datasets, this study provides the first comprehensive analysis of evidence for the spread of domestic animals across Neolithic central and western Turkey. We show that this process did not involve a set ‘package’ of domestic livestock but was instead more complex, likely involving multiple routes and pulses as well as interaction with local wild fauna along the way. These faunal patterns fit surprisingly well with current models for the westward expansion of Neolithic traditions [5], [6], [47], [108] and suggest that Aceramic and Ceramic Neolithic packages of distinctive material culture (including DFBW and RSBW traditions) were also characterized by unique animal economies.

In addition, the results of this project emphasize the benefits of new models of open data [13] and online data publication, which facilitate the replication and expansion of this study. Archaeologists have a long history of modeling cultural changes but rarely have they based regional syntheses on analytically replicable primary datasets. The approach advocated here is the first of its kind involving the large-scale, digital publication and integration of archaeozoological datasets and provides a template for future archaeological collaborations.

## Supporting Information

Figure S1 **Plots showing the frequencies of juvenile** ***Ovis, Capra, Bos*** **and** ***Sus*** **in assemblages through time based on epiphyseal fusion (see Text for explanation; see Tables S3-6 for data).** %Juvenile increases through time for all taxa except *Sus* (see Table S11 for results of Spearman Rank Correlation and associated p values). Colors reflect geographic location of site (after Fig. 1). “W” indicates assemblages representing wild populations. Points to the left of the vertical axis represent mean values for each region. For *Capra*, dark blue marks represent Zagros sites Asiab, ZC Shanidar, and Shanidar Mousterian.
(DOC)

Table S1 **List of sites used in this paper including phasing, chronologies, sample sizes, authors, and links to online databases where available.** Assemblages in bold were part of this data sharing project. Only biometric data from the Pendik and Yenikapı assemblages were included in this project. Assemblages in regular typeface represent previously published data.
(DOCX)

Table S2 **Relative abundance of** ***Ovis*** **,** ***Capra*** **,** ***Bos*** **and** ***Sus*** **in faunal assemblages based on NISP.**
(DOCX)

Table S3 **Mean and standard deviations of LSI values and % Juvenile for** ***Ovis*** **.**
(DOCX)

Table S4 **Mean and standard deviations of LSI values and % Juvenile for** ***Capra*** **.**
(DOCX)

Table S5 **Mean and standard deviations of LSI values and % Juvenile for** ***Bos*** **.**
(DOCX)

Table S6 **Mean and standard deviations of LSI values and % Juvenile for** ***Sus*** **.**
(DOCX)

Table S7 **Results of ANOVA for LSI values for** ***Ovis*** **.**
(XLSX)

Table S8 **Results of ANOVA for LSI values for** ***Capra*** **.**
(XLSX)

Table S9 **Results of ANOVA for LSI values for** ***Bos*** **.**
(XLSX)

Table S10 **Results of ANOVA for LSI values for** ***Sus*** **.**
(XLSX)

Table S11 **Spearman Rank Correlation tests for the relationship between A: taxonomic abundance and % juvenile and B: % juvenile and mean size (LSI), and C: % juvenile x time (yrs cal BC).**
(DOCX)

File S1 **Zooarchaeology at Bademağacı.** This file contains Table A-Table E. Table A, NISP data. Table B, Tooth eruption and wear data for *Ovis*/*Capra*, *Sus* and *Bos*. Table C, Biometric data for *Bos*. Table D, Biometric data for *Sus*. Table E, Biometric data for *Ovis* and *Capra*.
(XLS)

File S2 **LSI values.** This file contains Table F-Table I. Table F, LSI values for *Ovis*. Table G, LSI values for *Capra*. Table H, LSI values for *Bos*. Table I, LSI values for *Sus*.
(XLSX)

Alternative Language Abstract S1 **Turkish language abstract.**
(DOCX)

Text S1 **Methods of Data sharing and publication.**
(DOCX)
