# Correction: Data Sharing Reveals Complexity in the Westward Spread of Domestic Animals across Neolithic Turkey

**DOI:** 10.1371/journal.pone.0107824

**Published:** 2014-09-19

**Authors:** The PLOS ONE Staff

The first, second and last links in the Data Availability Statement are incorrect. The correct Data Availability Statement is:

The authors confirm that all data underlying the findings are fully available without restriction. Datasets used in this study have been deposited at opencontext.org and can be found at the following DOIs:

http://dx.doi.org/10.6078/M76H4FBS
http://doi.org/10.6078/M7VX0DF7
http://dx.doi.org/10.6078/M74Q7RW8
http://dx.doi.org/10.6078/M7X34VD1
http://dx.doi.org/10.6078/M7SB43PP
http://dx.doi.org/10.6078/M70Z715B
http://dx.doi.org/10.6078/M7KS6PHV
http://dx.doi.org/10.6078/M7D798BQ
http://dx.doi.org/10.6078/M7CC0XMT
http://dx.doi.org/10.6078/M73X84KX
http://dx.doi.org/10.6078/M7S46PVN
http://dx.doi.org/10.6078/M76H4FBS
http://dx.doi.org/10.6078/M78G8HM0.

The DOIs are incorrect in the Illipinar and Çatalhöyük Area TP site rows of Table S1. Please view the correct Table S1 here.

## Supporting Information

Table S1 **List of sites used in this paper including phasing, chronologies, sample sizes, authors, and links to online databases where available.** Assemblages in bold were part of this data sharing project. Only biometric data from the Pendik and Yenikapı assemblages were included in this project. Assemblages in regular typeface represent previously published data. doi:10.1371/journal.pone.0099845.s002
(DOCX)
