# Supplementary material for: Data Sharing Reveals Complexity in the Westward Spread of Domestic Animals across Neolithic Turkey
Source: PLoS One. 2014 Jun 13;9(6):e99845. doi: 10.1371/journal.pone.0099845 (PMC4057358; doi:10.1371/journal.pone.0099845)
Supplement: Figure S1 — Plots showing the frequencies of juvenile Ovis, Capra, Bos and Sus in assemblages through time based on epiphyseal fusion (see Text for explanation; see Tables S3-6 for data). %Juvenile increases through time for all taxa except Sus (see Table S11 for results of Spearman Rank Correlation and associated p values). Colors reflect geographic location of site (after Fig. 1). “W” indicates assemblages representing wild populations. Points to the left of the vertical axis represent mean values for each region. For Capra, dark blue marks represent Zagros sites Asiab, ZC Shanidar, and Shanidar Mousterian. (DOC) [file pone.0099845.s001.doc]

Supplementary Figure

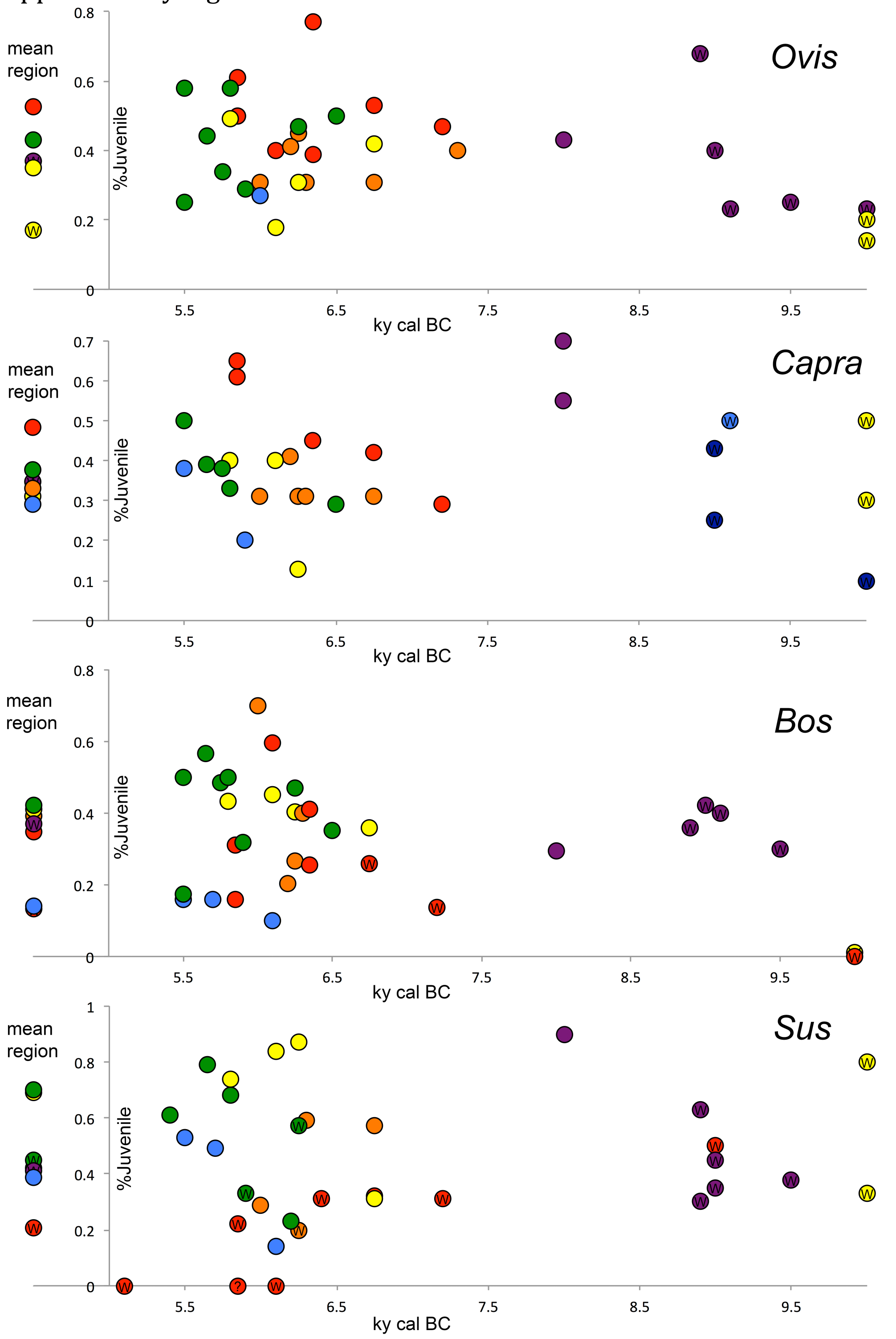

Figure S1. Plots showing the frequencies of juvenile *Ovis*, *Capra*, *Bos* and *Sus* in assemblages through time based on epiphyseal fusion (see Text for explanation; see Tables S3-6 for data). %Juvenile increases through time for all taxa except *Sus* (see Table S11 for results of Spearman Rank Correlation and associated p values). Colors reflect geographic location of site (after Fig. 1). "W" indicates assemblages representing wild populations. Points to the right of the vertical axis represent mean values for each region. For *Capra*, dark blue marks represent Zagros sites Asiab, ZC Shanidar, and Shanidar Mousterian.
