# Supplementary material for: Data Sharing Reveals Complexity in the Westward Spread of Domestic Animals across Neolithic Turkey
Source: PLoS One. 2014 Jun 13;9(6):e99845. doi: 10.1371/journal.pone.0099845 (PMC4057358; doi:10.1371/journal.pone.0099845)
Supplement: Table S2 — Relative abundance of Ovis , Capra , Bos and Sus in faunal assemblages based on NISP. (DOCX) [file pone.0099845.s003.docx]

| **Site** | **NISP** | ***Total Ovis/Capra+Ovis+Capra*** | ***Ovis/Capra*** | ***Ovis*** | ***Capra*** | ***Bos*** | ***Sus*** | **Other** | **Reference** |
| --- | --- | --- | --- | --- | --- | --- | --- | --- | --- |
| Uçağızlı | 40 | 35 | 0 | 0 | 35 | 0 | 5 | 126 | [1] |
| Hallan Çemi | 1491 | 939 | 579 | 322 | 38 | 3 | 549 | 695 | [2] |
| Körtik Tepe | 169 | 123 | 73 | 47 | 3 | 36 | 10 | 76 | [3] |
| Mureybet Natufian | 58 | 28 | 0 | 28 | 0 | 26 | 4 | 1071 | [4] |
| Mureybet PPNA | 1122 | 97 | 0 | 97 | 0 | 832 | 193 | 9161 | [4] |
| Göbekli | 4674 | 1237 | 944 | 293 | 0 | 2574 | 863 | 11838 | [5] |
| Direkli | 453 | 447 | 301 | 11 | 135 | 0 | 6 | 64 | [6] |
| Cafer | 3189 | 1731 | 877 | 272 | 582 | 418 | 1040 | 427 | [7] |
| Karain1 | 18659 | 18645 | 17744 | 621 | 280 | 7 | 7 | 81 | this project |
| Karain 2 | 7191 | 7189 | 6931 | 201 | 57 | 0 | 2 | 11 | this project |
| Öküzini 1 | 3044 | 3022 | 2426 | 397 | 199 | 0 | 22 | 1209 | this project |
| Öküzini 2 | 16003 | 16003 | 14244 | 1401 | 358 | 0 | 0 | 315 | this project |
| Öküzini 3 | 13662 | 13568 | 12241 | 1077 | 250 | 0 | 94 | 1032 | this project |
| Öküzini 4 | 1808 | 1808 | 1518 | 193 | 97 | 0 | 0 | 254 | this project |
| Öküzini 5 | 817 | 776 | 613 | 71 | 92 | 0 | 41 | 396 | this project |
| Boncuklu (%) | 79 | 4 | - | - | - | 33 | 42 | 12 | L. Martin |
| Aşıklı | 18674 | 16596 | 12123 | 3808 | 665 | 1738 | 340 | 899 | [8] |
| Musular (%) | 97 | 39 | - | - | - | 57 | 1 | 3 | [9] |
| Çatalhöyük Early | 3078 | 2322 | 1993 | 274 | 55 | 564 | 192 | 471 | this project |
| Çatalhöyük Middle | 5972 | 4273 | 3502 | 654 | 117 | 1336 | 363 | 1180 | this project |
| Çatalhöyük Late | 30866 | 27348 | 22200 | 4328 | 820 | 3103 | 415 | 1771 | this project |
| Çatalhöyük TP | 6344 | 5268 | 4404 | 716 | 148 | 989 | 87 | 312 | this project |
| Çatalhöyük West | 6865 | 6500 | 4604 | 1604 | 292 | 342 | 23 | 293 | this project |
| Güvercinkayasi | 1592 | 1451 | 752 | 566 | 133 | 113 | 28 | 143 | [10] |
| Köşk EC | 2322 | 1964 | 1207 | 582 | 175 | 340 | 18 | 860 | this project |
| Köşk MC | 2184 | 2028 | 1244 | 595 | 189 | 152 | 4 | 250 | this project |
| Suberde (%) | 96 | 83 | - | - | - | 5 | 8 | 4 | [11] |
| Erbaba III-I | 3570 | 3007 | 1862 | 936 | 209 | 216 | 347 | 309 | this project |
| Pinarbaşı B | 1446 | 1202 | 233 | 954 | 15 | 230 | 14 | 578 | this project |
| Pinarbaşı A | 65 | 39 | 27 | 12 | 0 | 12 | 14 | 67 | this project |
| Bademağacı ENI | 1508 | 851 | 732 | 80 | 39 | 341 | 316 | 79 | this project |
| Bademağacı ENII | 4198 | 2492 | 2023 | 283 | 186 | 899 | 807 | 299 | this project |
| Höyücek | 324 | 154 | 107 | 30 | 17 | 112 | 58 | 118 | [12] |
| Ulucak VI | 2236 | 1717 | 1504 | 168 | 45 | 353 | 166 | 81 | this project |
| Ulucak V | 4432 | 2815 | 2463 | 236 | 116 | 788 | 829 | 125 | this project |
| Ulucak IV | 2052 | 1357 | 1142 | 143 | 72 | 338 | 357 | 229 | this project |
| Çukurici | 1048 | 558 | 508 | 22 | 28 | 283 | 207 | 53 | this project |
| Yumuktepe | 206 | 158 | 146 | 4 | 8 | 35 | 13 | 3 | [13] |
| Domuztepe | 7770 | 3981 | 3339 | 317 | 325 | 2041 | 1748 | 83 | this project |
| Fikirtepe | 5575 | 2135 | 1488 | 523 | 124 | 3327 | 113 | 524 | [14] |
| Barcın | 2926 | 1582 | 1432 | 116 | 34 | 1277 | 67 | 47 | this project |
| Menteşe Early | 102 | 17 | 12 | 4 | 1 | 83 | 2 | 3 | this project |
| Menteşe Middle | 995 | 496 | 282 | 199 | 15 | 476 | 23 | 29 | this project |
| Menteşe Late | 870 | 580 | 364 | 196 | 20 | 268 | 22 | 3 | this project |
| Ilipinar X | 428 | 311 | 231 | 55 | 25 | 52 | 65 | 61 | this project |
| Ilipinar IX | 5662 | 3051 | 2243 | 355 | 453 | 910 | 1701 | 981 | this project |
| Ilipinar VIII | 6645 | 3632 | 2479 | 446 | 707 | 944 | 2069 | 404 | this project |
| Ilipinar VI-IV | 2162 | 1142 | 969 | 100 | 73 | 592 | 428 | 334 | this project |
| Çayönü Grill | 461 | 84 | 27 | 35 | 22 | 66 | 311 | 92 | [15] |
| Yeni Mahalle | 71 | 8 | 8 | 0 | 0 | 43 | 20 | 171 | [16] |
| Hasankeyf (%) | 73 | 60 | 60 | - | - | 1 | 12 | 2 | H.Hongo |
| Çayönü Round | 474 | 55 | 39 | 14 | 2 | 135 | 284 | 98 | (18) |
| Orman Fidanlğı | 1795 | 1544 | 1317 | 198 | 29 | 144 | 107 | 132 | [17] |
| Hoca Çesme | 811 | 589 | 520 | 17 | 52 | 176 | 46 | 26 | [18] |

Table S2. Relative abundance of *Ovis*, *Capra*, *Bos* and *Sus* in faunal assemblages based on NISP.

References Cited:

1. Kuhn SL, Stiner MC, Guleç E, Ozer I, Yilmaz H, et al. (2009) The early Upper Paleolithic occupations at Uçagızlı Cave (Hatay, Turkey). Journal of Human Evolution 56: 87-113.

2. Starkovich BM, Stiner MC (2009) Hallan Çemi Tepesi: High-ranked game exploitation alongside intensive seed processing at the Epipaleolithic-Neolithic transition in southeastern Turkey. Anthropozoologica 44: 41-62.

3. Arbuckle BS, Özkaya V (2007) Animal exploitation at Körtik Tepe: An early Aceramic Neolithic site in southeastern Turkey. Paléorient 32: 198-211.

4. Gourichon L, Helmer D (2008) Étude archéozoologique de Mureybet. In: Ibánez JJ, editor. Le site néolithique de Tell Mureybet (Syrie du Nord). Oxford: BAR International Series 1843. pp. 115-228.

5. von den Driesch A, Peters J (1999) Vorläufiger Bericht über die archäozoologischen Untersuchungen am Göbekli Tepe und am Gürcütepe bei Urfa, Türkei. Istanbuler Mitteilungen 49: 23-39.

6. Arbuckle BS, Erek CM (2012) Late Epipaleolithic hunters of the central Taurus: faunal remains from Direkli cave, Kahramanmaras, Turkey. International Journal of Osteoarchaeology 22: 694-707.

7. Helmer D (2008) Revision de la faune de Cafer Hoyuk (Malatya, Turquie): apports des methodes de l'analyse des melanges et de l'analyse de Kernel a la mise en evidence de la domestication. In: Vila E, Gourichon L, Choyke A, Buitenhuis H, editors. Archaeozoology of the Near East VIII. Lyon: Maison de l'Orient et de la Mediterranee. pp. 169-196.

8. Buitenhuis H (1997) Asıklı Höyük: A ‘protodomestication’ site. Anthropozoologica 25-26: 655-662.

9. Duru G, Özbaşaran M (2005) A 'non-domestic' site in central Anatolia. Anatolia Antiqua 8: 15-28.

10. Arbuckle BS, Öztan A, Gulçur S (2009) The evolution of sheep and goat husbandry in central Anatolia. Anthropozoologica 44: 129-157.

11. Perkins DP, Daly P (1968) A hunters' village in Neolithic turkey. Scientific American 219: 96-106.

12. De Cupere B, Duru R (2003) Faunal remains from Neolithic Höyücek (SW-Turkey) and the presence of early domestic cattle in Anatolia. Paléorient 29: 107-120.

13. Buitenhuis H, Caneva I (1998) Early animal breeding in south-eastern Anatolia: Mersin-Yumuktepe. In: Anreiter P, Bartosiewicz L, Jerem E, Meids W, editors. Man and the animal world. Budapest: Archaeolingua. pp. 122-130.

14. Boessneck J, von den Driesch A (1979) Die Tierknochenfunde aus der Neolithischen Siedlung auf dem Fikirtepe bei Kadiköy am Marmara Meer. München: Institut für Palaeoanatomie, Domestikationsforschung und Geschichte der Tiermedizin der Universität München.

15. Hongo H, Meadow RH, Öksüz B, Gülçin I (2004) Animal exploitation at Çayönü Tepesi, southeastern Anatolia. TÜBA-AR 7: 107-119.

16. Çelik B (2011) Sanlıurfa - Yeni Mahalle. In: Özdoğan M, Başgelen N, Kuniholm P, editors. The Neolithic in Turkey: The Euphrates Basin. Istanbul: Archaeology and Art Publications. pp. 139-164.

17. Uerpmann H-P (2001) Remarks on faunal remains from the Chalcolithic sites "Orman Fidanlığı" and "Kes Kaya" near Eskişehir in North-Western Anatolia. In: Efe T, editor. The salvage excavations at Orman Fidanlıgı: A Chalcolithic site in inland northwestern Anatolia. Istanbul: TASK Vakfı Yayınları. pp. 187-210.

18. Buitenhuis H (1996) Archaeozoology of the Holocene in Anatolia: a review. In: Demirci S, Ozer AM, Summers GD, editors. Archaemetry 94 Proceedings of the 29th International Symposium on Archaeometry, Ankara 1994. Ankara: Tubitak. pp. 411-420.
