# Supplementary material for: Data Sharing Reveals Complexity in the Westward Spread of Domestic Animals across Neolithic Turkey
Source: PLoS One. 2014 Jun 13;9(6):e99845. doi: 10.1371/journal.pone.0099845 (PMC4057358; doi:10.1371/journal.pone.0099845)
Supplement: Table S3 — Mean and standard deviations of LSI values and % Juvenile for Ovis . (DOCX) [file pone.0099845.s004.docx]

| **Site** | **LSI mean** | **sd** | **N (LSI)** | **%Juvenile** | **N (%Juv)** | **Author** |
| --- | --- | --- | --- | --- | --- | --- |
| Hallan Çemi | 0.0777 | 0.042 | 10 | 0.25 | - | [1] |
| Mureybet Natufian | 0.0299 | - | 8 | 0.23 | 21 | [2] |
| Mureybet PPNA | 0.0535 | - | 22 | 0.23 | 15 | [2] |
| Göbekli | 0.0666 | - | 85 | 0.40 | - | [3] |
| Cafer | 0.0281 | 0.025 | 21 | 0.43 | - | [4] |
| Karain | 0.0244 | 0.027 | 286 | 0.14 | 198 | Atici |
| Öküzini | 0.0088 | 0.025 | 214 | 0.20 | 149 | Atici |
| Aşıklı | 0.0346 | - | 12 | 0.38 | 1383 | [5,6] |
| Çatalhöyük Early | -0.0382 | 0.036 | 28 | +0.46 | - | Russell et al. |
| Çatalhöyük Middle | -0.0299 | 0.042 | 150 | 0.53 | 103 | Russell et al. |
| Çatalhöyük Late | -0.0329 | 0.035 | 1012 | 0.39 | 706 | Russell et al. |
| Çatalhöyük TP | -0.0439 | 0.041 | 139 | 0.40 | 5 | Marciniak |
| Çatalhöyük West | -0.0145 | 0.041 | 350 | 0.50 | 266 | Orton and Frame |
| Köşk EC | -0.0417 | 0.075 | 108 | 0.61 | 61 | Arbuckle |
| Suberde | 0.0197 | 0.05 | 42 | 0.40 | 29 | Arbuckle |
| Erbaba | -0.0028 | 0.043 | 393 | 0.45 | 369 | Arbuckle |
| Pinarbaşı B | -0.0521 | 0.06 | 39 | 0.77 | 81 | Carruthers |
| Bademağacı ENI | -0.0451 | 0.026 | 35 | +*0.24 | 21 | De Cupere |
| Bademağacı ENII | -0.0633 | 0.22 | 112 | +*0.37 | 176 | De Cupere |
| Bademağacı LN/EC | -0.0661 | 0.037 | 14 | +*0.25 | 68 | De Cupere |
| Höyücek | -0.0542 | 0.052 | 19 | +*0.41 | 40 | [7] |
| Ulucak VI | -0.0281 | 0.031 | 51 | 0.42 | 19 | Çakirlar |
| Uucak V | -0.0493 | 0.034 | 57 | 0.31 | 16 | Çakirlar |
| Uucak IV | -0.0578 | 0.031 | 94 | 0.49 | 45 | Çakirlar |
| Çukuriçi | -0.0476 | 0.05 | 11 | 0.18 | 11 | Galik |
| Yumuktepe | -0.0528 | 0.02 | 5 | - | - | [6] |
| Domuztepe I-III | -0.0399 | 0.035 | 172 | 0.27 | 72 | Kansa |
| Fikirtepe | -0.0435 | 0.034 | 82 | 0.50 | 96 | [8] |
| Barcın | -0.0663 | 0.039 | 46 | 0.47 | 34 | Galik |
| Menteşe Middle | -0.0656 | 0.033 | 84 | 0.58 | 26 | Gourichon and Helmer |
| Menteşe Late | -0.0735 | 0.034 | 83 | 0.58 | 40 | Gourichon and Helmer |
| Ilipinar X | -0.0345 | 0.035 | 29 | 0.29 | 14 | Buitenhuis |
| Ilipinar IX | -0.0343 | 0.037 | 353 | 0.34 | 155 | Buitenhuis |
| Ilipinar VIII | -0.0329 | 0.043 | 55 | 0.44 | 16 | Buitenhuis |
| Ilipinar VI-IV | -0.0525 | 0.035 | 68 | 0.25 | 40 | Buitenhuis |
| Pendik | -0.0353 | 0.021 | 21 | - | - | Peters and Pöllath |
| Orman Fidanlığı | -0.0316 | 0.027 | 125 | - | - | [9] |
| + based on teeth; * combined Ovis and Capra |  |  |  |  | |  |

Table S3. Mean and standard deviations of LSI values and % Juvenile (based on epiphyseal fusion) for *Ovis*.

References Cited:

1. Starkovich BM, Stiner MC (2009) Hallan Çemi Tepesi: High-ranked game exploitation alongside intensive seed processing at the Epipaleolithic-Neolithic transition in southeastern Turkey. Anthropozoologica 44: 41-62.

2. Gourichon L, Helmer D (2008) Étude archéozoologique de Mureybet. In: Ibánez JJ, editor. Le site néolithique de Tell Mureybet (Syrie du Nord). Oxford: BAR International Series 1843. pp. 115-228.

3. Peters J, von den Driesch A, Helmer D (2005) The upper Ephrates-Tigris basin: Cradle of agro-pastoralism? In: Vigne J-D, Peters J, Helmer D, editors. The first steps of animal domestication: New archaeological approaches Proceedings of the 9th ICAZ Conference, Durham 2002. Oxford: Oxbow. pp. 96-124.

4. Helmer D (2008) Revision de la faune de Cafer Hoyuk (Malatya, Turquie): apports des methodes de l'analyse des melanges et de l'analyse de Kernel a la mise en evidence de la domestication. In: Vila E, Gourichon L, Choyke A, Buitenhuis H, editors. Archaeozoology of the Near East VIII. Lyon: Maison de l'Orient et de la Mediterranee. pp. 169-196.

5. Buitenhuis H (1997) Asıklı Höyük: A ‘protodomestication’ site. Anthropozoologica 25-26: 655-662.

6. Buitenhuis H, Caneva I (1998) Early animal breeding in south-eastern Anatolia: Mersin-Yumuktepe. In: Anreiter P, Bartosiewicz L, Jerem E, Meids W, editors. Man and the animal world. Budapest: Archaeolingua. pp. 122-130.

7. De Cupere B, Duru R (2003) Faunal remains from Neolithic Höyücek (SW-Turkey) and the presence of early domestic cattle in Anatolia. Paléorient 29: 107-120.

8. Boessneck J, von den Driesch A (1979) Die Tierknochenfunde aus der Neolithischen Siedlung auf dem Fikirtepe bei Kadiköy am Marmara Meer. München: Institut für Palaeoanatomie, Domestikationsforschung und Geschichte der Tiermedizin der Universität München.

9. Uerpmann H-P (2001) Remarks on faunal remains from the Chalcolithic sites "Orman Fidanlığı" and "Kes Kaya" near Eskişehir in North-Western Anatolia. In: Efe T, editor. The salvage excavations at Orman Fidanlıgı: A Chalcolithic site in inland northwestern Anatolia. Istanbul: TASK Vakfı Yayınları. pp. 187-210.
