# Supplementary material for: Data Sharing Reveals Complexity in the Westward Spread of Domestic Animals across Neolithic Turkey
Source: PLoS One. 2014 Jun 13;9(6):e99845. doi: 10.1371/journal.pone.0099845 (PMC4057358; doi:10.1371/journal.pone.0099845)
Supplement: Table S4 — Mean and standard deviations of LSI values and % Juvenile for Capra . (DOCX) [file pone.0099845.s005.docx]

| **Site** | **LSI mean** | **sd** | **N (LSI)** | **%Juvenile** | **N (%Juv)** | **Author** |
| --- | --- | --- | --- | --- | --- | --- |
| Ksar Akil | 0.076 | 0.046 | 61 | - | - | [1] |
| Uçağızlı | 0.0365 | - | 154 | - | - | [2] |
| Hallan Çemi | 0.0522 | 0.043 | 6 | - | - | Arbuckle |
| Körtik | 0.012 | 0.013 | 3 | - | - | [3] |
| Direkli | 0.0595 | 0.044 | 35 | 0.50 | 110 | [4] |
| Cafer | 0.0357 | 0.023 | 25 | 0.55 | - | [5] |
| Karain | 0.0154 | 0.043 | 130 | 0.30 | 44 | Atici |
| Öküzini | -0.0042 | 0.041 | 58 | 0.50 | 26 | Atici |
| Aşıklı | 0.0323 | - | 7 | 0.45 | 269 | [6,7] |
| Çatalhöyük Early | -0.0459 | 0.029 | 3 | 0.29 | 7 | Russell et al. |
| Çatalhöyük Middle | -0.0446 | 0.031 | 19 | 0.42 | 12 | Russell et al. |
| Çatalhöyük Late | -0.0343 | 0.033 | 156 | 0.45 | 87 | Russell et al. |
| Çatalhöyük TP | - | - | - | 1.00 | 1 | Marciniak |
| Çatalhöyük West | -0.0296 | 0.036 | 77 | 0.65 | 34 | Orton and Frame |
| Köşk EC | -0.0417 | 0.075 | 40 | 0.61 | 28 | Arbuckle |
| Suberde | 0.0278 | 0.055 | 14 | - | - | Arbuckle |
| Erbaba | -0.0165 | 0.058 | 85 | 0.31 | 78 | Arbuckle |
| Bademağacı ENI | -0.0183 | 0.044 | 16 | +*0.24 | 21 | De Cupere |
| Bademağacı ENII | -0.0324 | 0.058 | 77 | +*0.37 | 176 | De Cupere |
| Bademağacı LN/EC | -0.0077 | 0.073 | 17 | +*0.25 | 68 | De Cupere |
| Höyücek | -0.0665 | 0.073 | 6 | +*0.41 | 40 | [8] |
| Ulucak VI | - | - | - | 0.17 | 6 | Çakirlar |
| Uucak V | -0.0524 | 0.036 | 35 | 0.13 | 15 | Çakirlar |
| Uucak IV | -0.0331 | 0.053 | 44 | 0.40 | 15 | Çakirlar |
| Çukuriçi | -0.0757 | 0.032 | 15 | 0.40 | 5 | Galik |
| Yumuktepe | -0.0471 | 0.025 | 4 | - | - | [7] |
| Domuztepe I-III | -0.0683 | 0.031 | 141 | 0.38 | 64 | Kansa |
| Fikirtepe | -0.0435 | 0.035 | 22 | 0.29 | 24 | [9] |
| Barcın | -0.0365 | 0.016 | 5 | 0.07 | 14 | Galik |
| Menteşe Middle | -0.049 | 0.015 | 5 | 0.33 | 3 | Gourichon and Helmer |
| Menteşe Late | -0.0546 | 0.043 | 8 | 0.50 | 7 | Gourichon and Helmer |
| Ilipinar X | -0.0477 | 0.035 | 12 | 0.20 | 5 | Buitenhuis |
| Ilipinar IX | -0.0555 | 0.035 | 437 | 0.38 | 206 | Buitenhuis |
| Ilipinar VIII | -0.0682 | 0.036 | 54 | 0.39 | 13 | Buitenhuis |
| Ilipinar VI-IV | -0.0733 | 0.034 | 33 | 0.15 | 13 | Buitenhuis |
| Pendik | -0.0381 | 0.032 | 5 | - | - | Peters and Pöllath |
| Asiab | 0.0307 | - | 25 | 0.43 | - | [10] |
| ZC Shanidar | 0.0447 | - | 20 | 0.25 | - | [11] |
| Shanidar Mousterian | 0.0233 | - | 26 | 0.10 | - | [12] |
| + based on teeth; * includes Ovis and Capra | |  |  |  |  |  |

Table S4. Mean and standard deviations of LSI values and % Juvenile (based on epiphyseal fusion) for *Capra*.

References Cited:

1. Kersten AMP (1987) Age and sex composition of Epipaleolithic fallow deer and wild goat from Ksar 'Akil. Palaeohistoria 29: 119-131.

2. Açıkkol A (2006) ÜçağIızlı Mağarası Faunasının Zooarkeolojik Açıdan Incelenmesi: Capra, Capreolus, Dama, ve Cervusların Morfometrik Açıdan Analizi [PhD Thesis]: Ankara University.

3. Arbuckle BS, Özkaya V (2007) Animal exploitation at Körtik Tepe: An early Aceramic Neolithic site in southeastern Turkey. Paléorient 32: 198-211.

4. Arbuckle BS, Erek CM (2012) Late Epipaleolithic hunters of the central Taurus: faunal remains from Direkli cave, Kahramanmaras, Turkey. International Journal of Osteoarchaeology 22: 694-707.

5. Helmer D (2008) Revision de la faune de Cafer Hoyuk (Malatya, Turquie): apports des methodes de l'analyse des melanges et de l'analyse de Kernel a la mise en evidence de la domestication. In: Vila E, Gourichon L, Choyke A, Buitenhuis H, editors. Archaeozoology of the Near East VIII. Lyon: Maison de l'Orient et de la Mediterranee. pp. 169-196.

6. Buitenhuis H (1997) Asıklı Höyük: A ‘protodomestication’ site. Anthropozoologica 25-26: 655-662.

7. Buitenhuis H, Caneva I (1998) Early animal breeding in south-eastern Anatolia: Mersin-Yumuktepe. In: Anreiter P, Bartosiewicz L, Jerem E, Meids W, editors. Man and the animal world. Budapest: Archaeolingua. pp. 122-130.

8. De Cupere B, Duru R (2003) Faunal remains from Neolithic Höyücek (SW-Turkey) and the presence of early domestic cattle in Anatolia. Paléorient 29: 107-120.

9. Boessneck J, von den Driesch A (1979) Die Tierknochenfunde aus der Neolithischen Siedlung auf dem Fikirtepe bei Kadiköy am Marmara Meer. München: Institut für Palaeoanatomie, Domestikationsforschung und Geschichte der Tiermedizin der Universität München.

10. Bökönyi S (1977) Animal remains from the Kermanshah valley, Iran. Oxford: BAR Supplement Series 34.

11. Zeder MA (2008) Animal domestication in the Zagros: an update and directions for future research. In: Vila E, Gourichon L, Choyke A, Buitenhuis H, editors. Archaeozoology of the Near East VIII. Lyon: Maison de l'Orient et de la Mediterranee. pp. 243-278.

12. Evins MA (1982) The fauna from Shanidar Cave: Mousterian wild goat exploitation in northeastern Iraq. Paléorient 8: 37-58.
