# Supplementary material for: Data Sharing Reveals Complexity in the Westward Spread of Domestic Animals across Neolithic Turkey
Source: PLoS One. 2014 Jun 13;9(6):e99845. doi: 10.1371/journal.pone.0099845 (PMC4057358; doi:10.1371/journal.pone.0099845)
Supplement: Table S5 — Mean and standard deviations of LSI values and % Juvenile for Bos . (DOCX) [file pone.0099845.s006.docx]

| **Site** | **LSI mean** | **sd** | **N (LSI)** | **%Juvenile** | **N (%Juv)** | **Author** |
| --- | --- | --- | --- | --- | --- | --- |
| Körtik Tepe | 0.0095 | 0.048 | 10 | 0.36 | 22 | [1] |
| Mureybet PPNA | -0.01 | 0.024 | 30 | 0.40 | 49 | [2] |
| Göbekli | 0.006 | - | 376 | 0.42 | - | [3] |
| Cafer | 0.0149 | - | 25 | 0.29 | - | [4] |
| Karain | 0.0678 | 0.0299 | 2 | 0.00 | 2 | Atici |
| Aşıklı | -0.01 | - | 131 | - | - | [5,6] |
| Musular | 0.01 | - | 316 | - | - | [5] |
| Çatalhöyük Early | 0.0239 | 0.035 | 33 | 0.14 | 95 | Russell et al. |
| Çatalhöyük Middle | 0.0228 | 0.04 | 86 | 0.26 | 296 | Russell et al. |
| Çatalhöyük Late | -0.0196 | 0.038 | 194 | 0.26 | 546 | Russell et al. |
| Çatalhöyük TP | -0.0464 | 0.052 | 53 | 0.59 | 32 | Marciniak |
| Çatalhöyük West | -0.052 | 0.052 | 27 | 0.31 | 71 | Orton and Frame |
| Köşk EC | -0.0417 | 0.075 | 164 | 0.16 | 252 | Arbuckle |
| Suberde | 0.021 | 0.006 | 2 | - | - | [7] |
| Erbaba | -0.038 | 0.046 | 126 | 0.26 | 209 | Arbuckle |
| Pinarbaşı A | - | - | - | 0.00 | 2 | Carruthers |
| Pinarbaşı B | -0.021 | 0.048 | 2 | 0.41 | 17 | Carruther |
| Bademağacı ENI | -0.0637 | 0.027 | 48 | - | - | De Cupere |
| Bademağacı ENII | -0.0664 | 0.029 | 213 | +0.4 | 10 | De Cupere |
| Bademağacı LN/EC | -0.0625 | 0.036 | 86 | +0.7 | 15 | De Cupere |
| Höyücek | -0.0527 | 0.035 | 76 | 0.2 | 64 | [8] |
| Ulucak VI | -0.0241 | 0.048 | 6 | 0.36 | 25 | Çakirlar |
| Uucak V | -0.0649 | 0.032 | 24 | 0.4 | 77 | Çakirlar |
| Uucak IV | -0.0631 | 0.021 | 22 | 0.43 | 74 | Çakirlar |
| Çukuriçi | -0.066 | 0.043 | 25 | 0.45 | 60 | Galik |
| Yumuktepe | -0.0661 | 0.006 | 4 | - | - | [9] |
| Domuztepe I | -0.069 | 0.043 | 361 | 0.16 | 613 | Kansa |
| Domuztepe II | -0.0433 | 0.035 | 18 | 0.16 | 57 | Kansa |
| Domuztepe III | -0.058 | 0.047 | 50 | 0.1 | 52 | Kansa |
| Fikirtepe | -0.0435 | 0.034 | 214 | +0.35 | 383 | [10] |
| Barcın | -0.0751 | 0.057 | 52 | 0.47 | 237 | Galik |
| Menteşe Early | -0.0535 | 0.045 | 9 | - | - | Gourichon and Helmer |
| Menteşe Middle | -0.0678 | 0.029 | 43 | 0.50 | 44 | Gourichon and Helmer |
| Menteşe Late | -0.0852 | 0.031 | 15 | 0.50 | 44 | Gourichon and Helmer |
| Ilipinar X | -0.0932 | 0.031 | 12 | 0.32 | 22 | Buitenhuis |
| Ilipinar IX | -0.0823 | 0.043 | 245 | 0.49 | 546 | Buitenhuis |
| Ilipinar VIII | -0.0994 | 0.033 | 16 | 0.56 | 39 | Buitenhuis |
| Ilipinar VI-IV | -0.096 | 0.046 | 223 | 0.17 | 317 | Buitenhuis |
| Pendik | -0.0467 | 0.038 | 35 | - | - | Peters and Pöllath |
| Orman Fidanlığı | -0.0677 | 0.0411 | 14 | - | - | [11] |
| + based on tooth eruption | |  |  |  |  |  |

Table S5. Mean and standard deviations of LSI values and % Juvenile (based on epiphyseal fusion) for *Bos*.

References Cited:

1. Arbuckle BS, Özkaya V (2007) Animal exploitation at Körtik Tepe: An early Aceramic Neolithic site in southeastern Turkey. Paléorient 32: 198-211.

2. Gourichon L, Helmer D (2008) Étude archéozoologique de Mureybet. In: Ibánez JJ, editor. Le site néolithique de Tell Mureybet (Syrie du Nord). Oxford: BAR International Series 1843. pp. 115-228.

3. Peters J, von den Driesch A, Helmer D (2005) The upper Ephrates-Tigris basin: Cradle of agro-pastoralism? In: Vigne J-D, Peters J, Helmer D, editors. The first steps of animal domestication: New archaeological approaches Proceedings of the 9th ICAZ Conference, Durham 2002. Oxford: Oxbow. pp. 96-124.

4. Helmer D (2008) Revision de la faune de Cafer Hoyuk (Malatya, Turquie): apports des methodes de l'analyse des melanges et de l'analyse de Kernel a la mise en evidence de la domestication. In: Vila E, Gourichon L, Choyke A, Buitenhuis H, editors. Archaeozoology of the Near East VIII. Lyon: Maison de l'Orient et de la Mediterranee. pp. 169-196.

5. Russell N, Martin L, Buitenhuis H (2005) Cattle domestication at Çatalhoyuk revisited. Current Anthropology 46 Supplement: S101-108.

6. Payne S (1985) Animal bones from Asikli Huyuk. Anatolian Studies 35: 109-122.

7. Perkins DP, Daly P (1968) A hunters' village in Neolithic turkey. Scientific American 219: 96-106.

8. De Cupere B, Duru R (2003) Faunal remains from Neolithic Höyücek (SW-Turkey) and the presence of early domestic cattle in Anatolia. Paléorient 29: 107-120.

9. Buitenhuis H, Caneva I (1998) Early animal breeding in south-eastern Anatolia: Mersin-Yumuktepe. In: Anreiter P, Bartosiewicz L, Jerem E, Meids W, editors. Man and the animal world. Budapest: Archaeolingua. pp. 122-130.

10. Boessneck J, von den Driesch A (1979) Die Tierknochenfunde aus der Neolithischen Siedlung auf dem Fikirtepe bei Kadiköy am Marmara Meer. München: Institut für Palaeoanatomie, Domestikationsforschung und Geschichte der Tiermedizin der Universität München.

11. Uerpmann H-P (2001) Remarks on faunal remains from the Chalcolithic sites "Orman Fidanlığı" and "Kes Kaya" near Eskişehir in North-Western Anatolia. In: Efe T, editor. The salvage excavations at Orman Fidanlıgı: A Chalcolithic site in inland northwestern Anatolia. Istanbul: TASK Vakfı Yayınları. pp. 187-210.
