# Supplementary material for: Data Sharing Reveals Complexity in the Westward Spread of Domestic Animals across Neolithic Turkey
Source: PLoS One. 2014 Jun 13;9(6):e99845. doi: 10.1371/journal.pone.0099845 (PMC4057358; doi:10.1371/journal.pone.0099845)
Supplement: Table S6 — Mean and standard deviations of LSI values and % Juvenile for Sus . (DOCX) [file pone.0099845.s007.docx]

| **Site** | **LSI mean** | **sd** | **N (LSI)** | **%Juvenile** | **N (%Juv)** | **Author** |
| --- | --- | --- | --- | --- | --- | --- |
| Hallan Çemi | 0.0218 | 0.015 | 5 | 0.38 | - | [1] |
| Körtik Tepe | 0.0471 | 0.051 | 3 | 0.63 | 8 | [2] |
| Mureybet PPNA | 0.0643 | 0.028 | 37 | 0.35 | 76 | [3] |
| Göbekli | 0.039 | - | 34 | - | - | [4] |
| Cafer | 0.0405 | 0.04 | 49 | 0.92 | - | [5] |
| Karain | - | - | - | 0.33 | 3 | Atici |
| Öküzini | -0.0009 | - | 1 | 0.80 | 5 | Atici |
| Aşıklı | 0.0333 | 0.033 | 7 | - | - | [6,7] |
| Pinarbaşı A | - | - | - | 0.50 | 2 | Carruthers |
| Çatalhöyük Early | 0.0304 | 0.05 | 5 | 0.31 | 39 | Russell et al. |
| Çatalhöyük Middle | 0.042 | 0.033 | 13 | 0.32 | 41 | Russell et al. |
| Çatalhöyük Late | 0.0409 | 0.039 | 15 | 0.31 | 59 | Russell et al. |
| Çatalhöyük TP | 0.0597 | 0.085 | 2 | 0.00 | 1 | Marciniak |
| Çatalhöyük West | -0.0452 | 0.023 | 2 | 0.00 | 2 | Orton and Frame |
| Köşk EC | 0.0299 | 0.026 | 9 | 0.22 | 9 | Arbuckle |
| Köşk MC | 0.094 | 0.04 | 4 | 0.00 | 2 | Arbuckle |
| Suberde | 0.0552 | 0.053 | 3 | - | - | [8] |
| Erbaba III-I | 0.0329 | - | 51 | 0.20 | 12 | Arbuckle, Dobney |
| Bademağacı ENI | -0.0568 | 0.077 | 11 | +0.57 | 12 | De Cupere |
| Bademağacı ENII | -0.0447 | 0.069 | 66 | +0.59 | 86 | De Cupere |
| Bademağacı LN/EC | -0.0561 | 0.078 | 9 | +0.29 | 14 | De Cupere |
| Höyücek | -0.0388 | 0.078 | 15 | 0.23 | 13 | [9] |
| Ulucak VI | -0.0745 | 0.004 | 2 | 0.31 | 45 | Çakirlar |
| Uucak V | -0.0622 | 0.036 | 15 | 0.87 | 87 | Çakirlar |
| Uucak IV | -0.0498 | 0.047 | 22 | 0.74 | 157 | Çakirlar |
| Çukuriçi | -0.0339 | 0.042 | 6 | 0.84 | 36 | Galik |
| Yumuktepe | -0.0328 | 0.036 | 3 | - | - | [10] |
| Domuztepe I | -0.0761 | 0.055 | 106 | 0.53 | 459 | Kansa |
| Domuztepe II | -0.0689 | 0.016 | 5 | 0.49 | 37 | Kansa |
| Domuztepe III | -0.0545 | 0.061 | 10 | 0.14 | 22 | Kansa |
| Fikirtepe | 0.0757 | 0.034 | 14 | - | - | [11] |
| Barcın | 0.0457 | - | 1 | 0.57 | 7 | Galik |
| Menteşe Early | 0.0146 | - | 1 | - | - | Gourichon and Helmer |
| Menteşe Late | -0.0969 | 0.028 | 3 | - | - | Gourichon and Helmer |
| Ilipinar X | 0.0165 | 0.078 | 13 | 0.33 | 27 | Buitenhuis |
| Ilipinar IX | -0.0693 | 0.061 | 143 | 0.68 | 1205 | Buitenhuis |
| Ilipinar VIII | -0.0703 | 0.054 | 164 | 0.79 | 131 | Buitenhuis |
| Ilipinar VII | -0.0696 | 0.042 | 33 | - | - | Buitenhuis |
| Ilipinar VI | -0.0877 | 0.072 | 9 | - | - | Buitenhuis |
| Ilipinar V | -0.0515 | 0.066 | 30 | 0.61 | 93 | Buitenhuis |
| Ilipinar IV | - | - | - | 0.48 | 23 | Buitenhuis |
| Pendik | -0.0129 | 0.017 | 4 | - | - | Çakırlar |
| Yenikapı | -0.0616 | 0.038 | 20 | - | - | Çakırlar |
| Orman Fidanlığı | -0.0995 | 0.049 | 12 | - | - | [12] |
| + based on tooth eruption |  |  |  |  |  |  |

Table S6. Mean and standard deviations of LSI values and % Juvenile (based on epiphyseal fusion) for *Sus*.

References Cited:

1. Rosenberg M, Nesbitt R, Redding R, Peasnall BL (1998) Hallan Çemi, pig husbandry, and post-Pleistocene adaptations along the Taurus-Zagros arc (Turkey). Paléorient 24: 25-41.

2. Arbuckle BS, Özkaya V (2007) Animal exploitation at Körtik Tepe: An early Aceramic Neolithic site in southeastern Turkey. Paléorient 32: 198-211.

3. Gourichon L, Helmer D (2008) Étude archéozoologique de Mureybet. In: Ibánez JJ, editor. Le site néolithique de Tell Mureybet (Syrie du Nord). Oxford: BAR International Series 1843. pp. 115-228.

4. Peters J, von den Driesch A, Helmer D (2005) The upper Ephrates-Tigris basin: Cradle of agro-pastoralism? In: Vigne J-D, Peters J, Helmer D, editors. The first steps of animal domestication: New archaeological approaches Proceedings of the 9th ICAZ Conference, Durham 2002. Oxford: Oxbow. pp. 96-124.

5. Helmer D (2008) Revision de la faune de Cafer Hoyuk (Malatya, Turquie): apports des methodes de l'analyse des melanges et de l'analyse de Kernel a la mise en evidence de la domestication. In: Vila E, Gourichon L, Choyke A, Buitenhuis H, editors. Archaeozoology of the Near East VIII. Lyon: Maison de l'Orient et de la Mediterranee. pp. 169-196.

6. Payne S (1985) Animal bones from Asikli Huyuk. Anatolian Studies 35: 109-122.

7. Buitenhuis H, Caneva I (1998) Early animal breeding in south-eastern Anatolia: Mersin-Yumuktepe. In: Anreiter P, Bartosiewicz L, Jerem E, Meids W, editors. Man and the animal world. Budapest: Archaeolingua. pp. 122-130.

8. Perkins DP, Daly P (1968) A hunters' village in Neolithic turkey. Scientific American 219: 96-106.

9. De Cupere B, Duru R (2003) Faunal remains from Neolithic Höyücek (SW-Turkey) and the presence of early domestic cattle in Anatolia. Paléorient 29: 107-120.

10. Buitenhuis H (2004) The importance of Yumuktepe in the origin and spread of animal domestication. In: Caneva I, Sevin V, editors. Mersin-Yumuktepe: A reappraisal. Lecce: Congedo Editore. pp. 163-168.

11. Boessneck J, von den Driesch A (1979) Die Tierknochenfunde aus der Neolithischen Siedlung auf dem Fikirtepe bei Kadiköy am Marmara Meer. München: Institut für Palaeoanatomie, Domestikationsforschung und Geschichte der Tiermedizin der Universität München.

12. Uerpmann H-P (2001) Remarks on faunal remains from the Chalcolithic sites "Orman Fidanlığı" and "Kes Kaya" near Eskişehir in North-Western Anatolia. In: Efe T, editor. The salvage excavations at Orman Fidanlıgı: A Chalcolithic site in inland northwestern Anatolia. Istanbul: TASK Vakfı Yayınları. pp. 187-210.
