# Supplementary material for: Data Sharing Reveals Complexity in the Westward Spread of Domestic Animals across Neolithic Turkey
Source: PLoS One. 2014 Jun 13;9(6):e99845. doi: 10.1371/journal.pone.0099845 (PMC4057358; doi:10.1371/journal.pone.0099845)
Supplement: Table S11 — Spearman Rank Correlation tests for the relationship between A: taxonomic abundance and % juvenile and B: % juvenile and mean size (LSI), and C: % juvenile x time (yrs cal BC). (DOCX) [file pone.0099845.s012.docx]

| **A. %NISP x %Juvenile** | **df** | **rs** | **p (one tailed)** | **p (two tailed)** |
| --- | --- | --- | --- | --- |
| *Ovis* | 30 | 0.2964 | **0.0497** | 0.0995 |
| *Capra* | 23 | 0.0085 | 0.4842 | 0.9684 |
| *Bos* | 26 | 0.1597 | 0.2098 | 0.4196 |
| *Sus* | 26 | 0.5083 | **0.0028** | **0.0057** |
| **B. %Juvenile x LSI mean** | **df** | **rs** | **p (one tailed)** | **p (two tailed)** |
| *Ovis* | 30 | -0.2798 | 0.06 | 0.12 |
| *Capra* | 24 | 0.0164 | 0.4684 | 0.9369 |
| *Bos* | 26 | -0.4741 | **0.0053** | **0.0107** |
| *Sus* | 26 | -0.2261 | 0.1243 | 0.2486 |
| **C. %Juvenile x Time (yrs cal BC)** | **df** | **rs** | **p (one tailed)** | **p (two tailed)** |
| *Ovis* | 30 | -0.3263 | **0.0342** | 0.0684 |
| *Capra* | 24 | -0.197 | 0.1684 | 0.3368 |
| *Bos* | 26 | -0.2419 | 0.1076 | 0.2153 |
| *Sus* | 26 | 0.1087 | 0.2901 | 0.5802 |

Table S11. Spearman Rank Correlation tests for the relationship between A. taxonomic abundance and % juvenile and B. % juvenile and mean size (LSI), and C. % juvenile x time (yrs cal BC).
