# Supplementary material for: Data Sharing Reveals Complexity in the Westward Spread of Domestic Animals across Neolithic Turkey
Source: PLoS One. 2014 Jun 13;9(6):e99845. doi: 10.1371/journal.pone.0099845 (PMC4057358; doi:10.1371/journal.pone.0099845)
Supplement: Alternative Language Abstract S1 — Turkish language abstract. (DOCX) [file pone.0099845.s015.docx]

Alternative Language Abstract S1 (Türkçe)

Özet

Bu çalışma Neolitik Türkiye’deki evcilleştirilmis havyanların ilk yayılımını belgelemek amacıyla, Epipaleolitik ile Kalkolitik dönemler arası, c. 18,000-4,000 cal BC, 17 kazıdan çıkarılmış 200,000’den fazla kemiğin temel arkeozooloji verilerini biraraya getiren bir bilgi entegrasyon projesinin sonuçlarını sunar. Bu çalişmada, Neolitik yaşam sürdürme teknolojilerinin batıya doğru yayılımının, koyun, keçi, sığır ve domuzdan oluşan hazır bir ‘paketi’ takip etmek yerine, birden fazla rotayı ve atılımı birleştirdiğini ispat ediyoruz. Bu çalişmadaki Neolitik hayvan ekonomilerinin, nicelik bakımından daha kısıtlı olan önceki araştırmalarla karşılaştırıldığında, çok daha çeşitli oldukları görülmüştür. Ayrıca, agro-pastoral ekonomilere geçiş sırasında, evcilleştirilmiş hayvanlar ve yerel vahşi fauna arasındaki etkileşimler devam etmistir. Bu proje, Open Context’te (opencontext.org) veri setlerini yayınlayarak, veri paylaşımının ve ana veri setlerinin web tabanli yayılımının arkeolojideki büyük araştırmalara faydasını vurgular.
