# Supplementary material for: Data Sharing Reveals Complexity in the Westward Spread of Domestic Animals across Neolithic Turkey
Source: PLoS One. 2014 Jun 13;9(6):e99845. doi: 10.1371/journal.pone.0099845 (PMC4057358; doi:10.1371/journal.pone.0099845)
Supplement: Text S1 — Methods of Data sharing and publication. (DOCX) [file pone.0099845.s016.docx]

SUPPORTING INFORMATION (SI)

**Text S1**

*Data sharing and publication*

Large-scale (for this discipline) data sharing and integration made this analysis possible. Data sharing took place within the context of the *Central and Western Anatolian Neolithic Animal Economies Working Group*. This group of researchers met at the Royal Belgian Institute of Natural Sciences, Brussels, in June 2011 in order to discuss and plan data sharing and then again in April, 2013 at Christian-Albrechts University, Kiel, where shared faunal datasets were collaboratively analyzed, interpreted and discussed by the authors.

The datasets used in this study were created in the context of separate, independent research programs. Before the individual datasets could be integrated and compared, they needed significant pre-processing, including data cleaning, documentation, and annotation with controlled vocabularies and ontologies (formal conceptual models used in data integration) using the conventions of “Linked Open Data” (LOD). LOD methods center on using Web URIs to identify shared concepts. The approach makes it easier to relate and integrate datasets and vocabularies curated by multiple expert communities working across the Web. For this study, we annotated zooarchaeological datasets with biological taxa concepts curated by the Encyclopedia of Life (EOL) and anatomical entities curated by UBERON for semantic alignment.

Data cleaning and annotation involve specialized expertise and tools not widely possessed among the participating researchers. Open Context’s “Data Editors” (SWK and EK) provided these informatics support services working in consultation with the data contributors to troubleshoot and resolve various issues. Once the datasets were prepared and integrated, they were circulated among the contributing researchers for study. All datasets are now available as open access data publications in Open Context (Table S1, with links to the projects). Because data editing involved annotating key fields in all datasets to common terms, the data could be grouped into three large integrated tables for analysis by the various working group members. The final data tables amounted to more than 200,000 archaeofaunal specimens representing seventeen sites, authored by eighteen researchers. Analysis included these 200,000+ records, as well as additional data from published sources (see Table S1 for list of sites). Details of the approaches used in data cleaning, documentation, semantic annotation and collaboration with data contributors are described elsewhere (1).

This study represents a landmark in demonstrating valuable insights gained from large-scale zooarchaeological data integration. It also demonstrates an innovative approach to promoting data sharing and reuse in the research community. Considering data sharing as a form of publishing can help create the reward structures that make data dissemination and reuse more scientifically and professionally rewarding (1). Recognition of data as a form of publication can align with the research community’s conventions and traditions (2), and may complement impact advantages sometimes observed on articles associated with open data (3).

References Cited:

1. Kansa EC & Kansa SW (2013) We All Know That a 14 Is a Sheep: Data Publication and Professionalism in Archaeological Communication. *Journal of Eastern Mediterranean Archaeology and Heritage Studies* 1(1):88-97.

2. Costello MJ (2009) Motivating Online Publication of Data. *BioScience* 59:418-427.

3. Piwowar H & Vision TJ (2013) Data Reuse and the Open Data Citation

Advantage. *PeerJ* 1:e175 <http://dx.doi.org/10.7717/peerj.175>
