# Supplementary material for: Correction: Data Sharing Reveals Complexity in the Westward Spread of Domestic Animals across Neolithic Turkey
Source: PLoS One. 2014 Sep 19;9(9):e107824. doi: 10.1371/journal.pone.0107824 (PMC4169568; doi:10.1371/journal.pone.0107824)
Supplement: Table S1 — List of sites used in this paper including phasing, chronologies, sample sizes, authors, and links to online databases where available. Assemblages in bold were part of this data sharing project. Only biometric data from the Pendik and Yenikapı assemblages were included in this project. Assemblages in regular typeface represent previously published data. doi:10.1371/journal.pone.0099845.s002 (DOCX) [file pone.0107824.s001.docx]

| **Site** | **Phases** | **Region** | **ky cal BC** | **# specimens** | **Author/Reference** | **Link to dataset** |
| --- | --- | --- | --- | --- | --- | --- |
| **Total dataset (NISP, fusion)** |  |  | 18-4.7 | 237,905 | [1] | http://dx.doi.org/10.6078/M7GQ6VPJ |
| **Total dataset (biometrics)** |  |  | 18-4.7 | 20,913 | [2] | http://dx.doi.org/10.6078/M7BZ63Z6 |
| **Çatalhöyük** | Total | Central | 7.4-5.7 | 145,642 | [3] | <http://dx.doi.org/10.6078/M7V985ZW> |
| **Çatalhöyük** | Early (KOPAL, South G) | Central | 7.4-7 | 14,173 | [3] | <http://dx.doi.org/10.6078/M7V985ZW> |
| **Çatalhöyük** | Middle (South H-N) | Central | 7-6.5 | 20,118 | [3] | <http://dx.doi.org/10.6078/M7V985ZW> |
| **Çatalhöyük** | Late (4040, North Area, South P-T) | Central | 6.5-6.2 | 76,983 | [3] | <http://dx.doi.org/10.6078/M7V985ZW> |
| **Çatalhöyük** | TP Area | Central | 6.3-6 | 14,974 | [4] | <http://doi.org/10.6078/M7VX0DF7> |
| **Çatalhöyük** | West Mound | Central | 6-5.7 | 13,866 | [3] | <http://dx.doi.org/10.6078/M7V985ZW> |
| **Köşk Höyük** | EC | Central | 6.2-5.5 | 5547 | [5] | <http://dx.doi.org/10.6078/M74Q7RW8> |
| **Köşk Höyük** | MC | Central | 5.4-4.7 | NA | [5] | <http://dx.doi.org/10.6078/M74Q7RW8> |
| **Pinarbaşı** | A | Central | 9 | 294 | [6] | <http://dx.doi.org/10.6078/M7X34VD1> |
| **Pinarbaşı** | B | Central | 6.7-6 | 3071 | [6] | <http://dx.doi.org/10.6078/M7X34VD1> |
| Boncuklu | Aceramic | Central | 8.5-7.5 | NA | [7] | NA |
| Aşıklı Höyük | Aceramic | Central | 8.2-7.5 | NA | [8-10] | NA |
| Musular | Aceramic/PN | Central | 7.5-7 | NA | [11] | NA |
| Yumuktepe | PN | South | 6.7-6 | NA | [9] | NA |
| **Domuztepe** | I-III | South | 6.5-5.5 | 9498 | [12] | <http://dx.doi.org/10.6078/M7SB43PP> |
| Üçağızlı | Epipal | South | 17 | NA | [13,14] | NA |
| Direkli | Epipal | South | 9 | NA | [15] | NA |
| **Bademağacı** | Total | Lakes | 6.7-5.5 | 12,029 | De Cupere | Tables A-E in File S1 |
| **Bademağacı** | ENI | Lakes | 6.7 | 3552 | De Cupere | Tables A-E in File S1 |
| **Bademağacı** | ENII | Lakes | 6.5-6.2 | 7183 | De Cupere | Tables A-E in File S1 |
| **Bademağacı** | PN/Chalc | Lakes | 6-5.5 | 750 | De Cupere | Tables A-E in File S1 |
| Höyücek | Lower Levels | Lakes | 6.4-6.2 | NA | [16] | NA |
| Höyücek | Upper Levels | Lakes | 6.2-6 | NA | [16] | NA |
| **Suberde** | Aceramic | Lakes | 7.5-7 | 359 | [17] | <http://dx.doi.org/10.6078/M70Z715B> |
| **Erbaba Höyük** | III-I | Lakes | 6.5-6 | 7090 | [17] | <http://dx.doi.org/10.6078/M70Z715B> |
| **Ulucak** | VI | West | 7-6.4 | 2766 | [18] | <http://dx.doi.org/10.6078/M7KS6PHV> |
| **Ulucak** | V | West | 6.5-6 | 3126 | [18] | <http://dx.doi.org/10.6078/M7KS6PHV> |
| **Ulucak** | IV | West | 6-5.7 | 4547 | [18] | <http://dx.doi.org/10.6078/M7KS6PHV> |
| **Çukuriçi** | VIII | West | 6.2-6 | 1577 | [19] | <http://dx.doi.org/10.6078/M7D798BQ> |
| **Karain B** | Epipal | West | 18-17 | 5152 | [20] | <http://dx.doi.org/10.6078/M7CC0XMT> |
| **Öküzini** | Total | West | 17.8-11.7 | 6386 | [21] | <http://dx.doi.org/10.6078/M73X84KX> |
| **Öküzini** | 1-3 | West | 17.8-11 | 4803 | [21] | <http://dx.doi.org/10.6078/M73X84KX> |
| **Öküzini** | 4 | West | 12.5-12.1 | 711 | [21] | <http://dx.doi.org/10.6078/M73X84KX> |
| **Öküzini** | 5 | West | 11.9-11.7 | 872 | [21] | <http://dx.doi.org/10.6078/M73X84KX> |
| **Menteşe** | Total | NW | 6.5-5.5 | 3139 | [22] | <http://dx.doi.org/10.6078/M7S46PVN> |
| **Menteşe** | ancien | NW | 6.5 | 124 | [22] | <http://dx.doi.org/10.6078/M7S46PVN> |
| **Menteşe** | moyenne | NW | 6.3-6 | 1439 | [22] | <http://dx.doi.org/10.6078/M7S46PVN> |
| **Menteşe** | recent | NW | 6-5.5 | 1576 | [22] | <http://dx.doi.org/10.6078/M7S46PVN> |
| **Ilıpinar** | Total | NW | 6-5.4 | 36,881 | [23] | <http://dx.doi.org/10.6078/M76H4FBS> |
| **Ilıpinar** | X | NW | 6-5.8 | 967 | [23] | <http://dx.doi.org/10.6078/M76H4FB>S |
| **Ilıpinar** | IX | NW | 5.8-5.7 | 21,452 | [23] | <http://dx.doi.org/10.6078/M76H4FB>S |
| **Ilıpinar** | VIII | NW | 5.7-5.6 | 2295 | [23] | <http://dx.doi.org/10.6078/M76H4FB>S |
| **Ilıpinar** | VII, VI | NW | 5.6-5.4 | 135 | [23] | <http://dx.doi.org/10.6078/M76H4FB>S |
| **Ilıpinar** | V | NW | 5.5-5.4 | 6282 | [23] | <http://dx.doi.org/10.6078/M76H4FB>S |
| **Ilıpinar** | IV | NW | 5.5-5.4 | 835 | [23] | <http://dx.doi.org/10.6078/M76H4FB>S |
| **Barcın Höyük** | VI | NW | 6.5-6 | 3742 | [24] | <http://dx.doi.org/10.6078/M78G8HM0> |
| **Pendik** | PN | NW | 6.5-6 | NA | Çakırlar, Peters, Pöllath | Tables A-D in File S2 |
| **Yenikapı** | PN | NW | 6 | NA | Çakırlar | Table D in File S2 |
| Fikirtepe | PN | NW | 6.5-6 | NA | [25] | NA |
| Orman Fidanlığı | PN | NW | 5.5 | NA | [26] | NA |
| Hoca Çesme | PN | NW | 6.5-6 | NA | [27] | NA |
| Mureybet | Epipal-PPNA | SE | 11-9 | NA | [28] | NA |
| Cafer | PPNB | SE | 8.5-7.5 | NA | [29] | NA |
| Göbekli | PPNA | SE | 9 | NA | [30] | NA |
| Nevalı Çori | PPNB | SE | 8.5-7.5 | NA | [30,31] | NA |
| Körtik Tepe | PPNA | SE | 9 | NA | [32] | NA |
| Çayönü Round | PPNA | SE | 9 | NA | [33] | NA |
| Çayönü Grill | PPNA/Early PPNB | SE | 9-8.5 | NA | [33] | NA |
| Yeni Mahalle | PPNA | SE | 9 | NA | [34] | NA |
| Hasankeyf | PPNA | SE | 9 | NA | H. Hongo | NA |
| Hallan Çemi | PPNA | SE | 10 | NA | [35] | NA |
| Asiab | PPNA | Iran | 9 | NA | [36,37] | NA |
| Shanidar | Mousterian | Iran | 50 | NA | [38] | NA |
| ZC Shanidar | Aceramic | Iran | 9 | NA | [37] | NA |
| Ksar Akil | Epipal | Lebanon | 15 | NA | [39] | NA |

Table S1. List of sites used in this paper including phasing, chronologies, sample sizes, authors, and links to online databases where available. Assemblages in bold were part of this data-sharing project. Only biometric data from the Pendik and Yenikapı assemblages were included in this project. Assemblages in regular typeface represent previously published data.

Works Cited:

1. Arbuckle BS, Orton D, Buitenhuis H, Marciniak A, Atici AL, et al. (2014) EOL Computational Data Challenge: Primary Zooarchaeology Dataset Version 2. Open Context. <http://opencontext.org/tables/314adedf882421055fc215a56ba7a79b>, <http://dx.doi.org/10.6078/M7GQ6VPJ>

2. Arbuckle BS, Orton D, Buitenhuis H, Marciniak A, Atici AL, et al. (2014) EOL Computational Data Challenge: Zooarchaeology Metrics Dataset Version 2. Open Context. <http://opencontext.org/tables/def8fb9c9d7fdc1993db45b7350ca955>, <http://dx.doi.org/10.6078/M7BZ63Z6>

3. Russell N, Twiss K, Frame S, Yeomans L, Martin L, et al. (2013) Çatalhöyük Main Zooarchaeological Dataset. Open Context: Open Context. <http://opencontext.org/tables/4c98ccdee589f0e76c5fa7376ac2638e>, <http://dx.doi.org/10.6078/M7V985ZW>

4. Marciniak A, Pawlowska K, Russell N, Twiss K, Martin L, et al. (2013) Çatalhöyük Area TP Main Zooarchaeological Dataset. Open Context: Open Context. <http://opencontext.org/tables/84332d5b790ecfbbf9a341273ea8d142>, <http://dx.doi.org/10.6078/M7PK0D3R>

5. Arbuckle BS (2013) Köşk Höyük Faunal Data. Open Context: Open Context. <http://opencontext.org/tables/f8b79a9771ccf176508cd52845a590b2>, <http://dx.doi.org/10.6078/M7H12ZXT>

6. Carruthers D (2013) Pınarbaşı EOL Computational Data Challenge Revised Zooarchaeology Dataset. Open Context: Open Context. <http://opencontext.org/tables/2e995b59d5adf5b079fde6cea24b251d>, <http://dx.doi.org/10.6078/M7X34VD1>

7. Baird D (2012) The Late Epipaleolithic, Neolithic and Chalcolithic of the Anatolian Plateau, 13,000-4000 BC. In: Potts DT, editor. A Companion to the Archaeology of the Ancient Near East Volume I. Malden, Massachusetts: Wiley-Blackwell. pp. 431-465.

8. Buitenhuis H (1997) Asıklı Höyük: A ‘protodomestication’ site. Anthropozoologica 25-26: 655-662.

9. Buitenhuis H, Caneva I (1998) Early animal breeding in south-eastern Anatolia: Mersin-Yumuktepe. In: Anreiter P, Bartosiewicz L, Jerem E, Meids W, editors. Man and the animal world. Budapest: Archaeolingua. pp. 122-130.

10. Peters J, Buitenhuis H, Grupe G, Schmidt K, Pöllath N (2013) The long and winding road. Ungulate exploitation and domestication in early Neolithic Anatolia (10,000 - 7,000 cal BC). In: Colledge S, Connolly J, Dobney K, Manning K, Shennan S, editors. Origins and Spread of Domestic Animals in Southwest Asia and Europe. Walnut Creek, CA: Left Coast Press. pp. 83-114.

11. Russell N, Martin L, Buitenhuis H (2005) Cattle domestication at Çatalhoyuk revisited. Current Anthropology 46 Supplement: S101-108.

12. Kansa SW (2013) Domuztepe EOL Computational Data Challenge Revised Zooarchaeology Dataset. Open Context. <http://opencontext.org/tables/6354e1db8815494679677836472c639c>, <http://dx.doi.org/10.6078/M7SB43PP>

13. Açıkkol A (2006) ÜçağIızlı Mağarası Faunasının Zooarkeolojik Açıdan Incelenmesi: Capra, Capreolus, Dama, ve Cervusların Morfometrik Açıdan Analizi [PhD Thesis]: Ankara University.

14. Kuhn SL, Stiner MC, Guleç E, Ozer I, Yilmaz H, et al. (2009) The early Upper Paleolithic occupations at Uçagızlı Cave (Hatay, Turkey). Journal of Human Evolution 56: 87-113.

15. Arbuckle BS, Erek CM (2012) Late Epipaleolithic hunters of the central Taurus: faunal remains from Direkli cave, Kahramanmaras, Turkey. International Journal of Osteoarchaeology 22: 694-707.

16. De Cupere B, Duru R (2003) Faunal remains from Neolithic Höyücek (SW-Turkey) and the presence of early domestic cattle in Anatolia. Paléorient 29: 107-120.

17. Arbuckle BS (2013) Erbaba Höyük and Suberde Zooarchaeology Data. Open Context: Open Contex. <http://opencontext.org/tables/6979344c2aaed2d1454ef0bba7763127>, <http://dx.doi.org/10.6078/M7C8276W>

18. Çakırlar C (2013) Ulucak Höyük Main Zooarchaeological Dataset. Open Context: Open Context. <http://opencontext.org/tables/846e3cd8ad23c8b4ca5ed378b4e1672e>, <http://dx.doi.org/10.6078/M79884XQ>

19. Galik A (2013) Çukuriçi Höyük Zooarchaeology. Open Context: Open Context. <http://opencontext.org/tables/8e8708b1d72413f645688bad97cfca54>, <http://dx.doi.org/10.6078/M7D798BQ>

20. Atici AL (2013) Zooarchaeology of Karain Cave B Data. Open Context: Open Context. <http://opencontext.org/tables/6bd43fb3d4676cd4735f8132a225d9ae>, <http://dx.doi.org/10.6078/M7SF2T31>

21. Atici AL (2013) Zooarchaeology of Öküzini Cave Data. Open Context: Open Context. <http://opencontext.org/tables/bd3f57be6fcb11492827c112a467ca4c>, <http://dx.doi.org/10.6078/M7NP22CP>

22. Gourichon L, Helmer D (2013) Faunal Data from Neolithic Menteşe. <http://opencontext.org/tables/2d7ed24fb9bfce0b7085983d55930dc3>, <http://dx.doi.org/10.6078/M7MG7MD8>

23. Buitenhuis H (2013) Ilıpınar Zooarchaeology Main Zooarchaeological Dataset. Open Context: Open Context. <http://opencontext.org/tables/3746e117f8bd3c648568cdd59d00272c>, <http://dx.doi.org/10.6078/M72R3PM2>

24. Galik A (2013) Barçın Höyük Zooarchaeology Data. Open Context: Open Context. <http://opencontext.org/tables/23d7c8387a870c56fd4b5d47500f6311>, <http://dx.doi.org/10.6078/M7MS3QN7>

25. Boessneck J, von den Driesch A (1979) Die Tierknochenfunde aus der Neolithischen Siedlung auf dem Fikirtepe bei Kadiköy am Marmara Meer. München: Institut für Palaeoanatomie, Domestikationsforschung und Geschichte der Tiermedizin der Universität München.

26. Uerpmann H-P (2001) Remarks on faunal remains from the Chalcolithic sites "Orman Fidanlığı" and "Kes Kaya" near Eskişehir in North-Western Anatolia. In: Efe T, editor. The salvage excavations at Orman Fidanlıgı: A Chalcolithic site in inland northwestern Anatolia. Istanbul: TASK Vakfı Yayınları. pp. 187-210.

27. Buitenhuis H (1996) Archaeozoology of the Holocene in Anatolia: a review. In: Demirci S, Ozer AM, Summers GD, editors. Archaemetry 94 Proceedings of the 29th International Symposium on Archaeometry, Ankara 1994. Ankara: Tubitak. pp. 411-420.

28. Gourichon L, Helmer D (2008) Étude archéozoologique de Mureybet. In: Ibánez JJ, editor. Le site néolithique de Tell Mureybet (Syrie du Nord). Oxford: BAR International Series 1843. pp. 115-228.

29. Helmer D (2008) Revision de la faune de Cafer Hoyuk (Malatya, Turquie): apports des methodes de l'analyse des melanges et de l'analyse de Kernel a la mise en evidence de la domestication. In: Vila E, Gourichon L, Choyke A, Buitenhuis H, editors. Archaeozoology of the Near East VIII. Lyon: Maison de l'Orient et de la Mediterranee. pp. 169-196.

30. Peters J, von den Driesch A, Helmer D (2005) The upper Ephrates-Tigris basin: Cradle of agro-pastoralism? In: Vigne J-D, Peters J, Helmer D, editors. The first steps of animal domestication: New archaeological approaches Proceedings of the 9th ICAZ Conference, Durham 2002. Oxford: Oxbow. pp. 96-124.

31. von den Driesch A, Peters J (2001) Fruheste Haustierhaltung in der Sudostturkei. In: Boehmer RJ, Maran J, editors. Lux Orientis: Archaologie zwischen Asien und Europa. Rahden: M. Leidorf. pp. 113-120.

32. Arbuckle BS, Özkaya V (2007) Animal exploitation at Körtik Tepe: An early Aceramic Neolithic site in southeastern Turkey. Paléorient 32: 198-211.

33. Hongo H, Meadow RH, Öksüz B, Gülçin I (2004) Animal exploitation at Çayönü Tepesi, southeastern Anatolia. TÜBA-AR 7: 107-119.

34. Çelik B (2011) Sanlıurfa - Yeni Mahalle. In: Özdoğan M, Başgelen N, Kuniholm P, editors. The Neolithic in Turkey: The Euphrates Basin. Istanbul: Archaeology and Art Publications. pp. 139-164.

35. Starkovich BM, Stiner MC (2009) Hallan Çemi Tepesi: High-ranked game exploitation alongside intensive seed processing at the Epipaleolithic-Neolithic transition in southeastern Turkey. Anthropozoologica 44: 41-62.

36. Bökönyi S (1977) Animal remains from the Kermanshah valley, Iran. Oxford: BAR Supplement Series 34.

37. Zeder MA (2008) Animal domestication in the Zagros: an update and directions for future research. In: Vila E, Gourichon L, Choyke A, Buitenhuis H, editors. Archaeozoology of the Near East VIII. Lyon: Maison de l'Orient et de la Mediterranee. pp. 243-278.

38. Evins MA (1982) The fauna from Shanidar Cave: Mousterian wild goat exploitation in northeastern Iraq. Paléorient 8: 37-58.

39. Kersten AMP (1987) Age and sex composition of Epipaleolithic fallow deer and wild goat from Ksar 'Akil. Palaeohistoria 29: 119-131.
